# The divergence and dispersal of early perissodactyls as evidenced by early Eocene equids from Asia

**DOI:** 10.1038/s42003-018-0116-5

**Published:** 2018-08-15

**Authors:** Bin Bai, Yuan-Qing Wang, Jin Meng

**Affiliations:** 1 Key Laboratory of Vertebrate Evolution and Human Origins of Chinese Academy of Sciences, Institute of Vertebrate Paleontology and Paleoanthropology, Chinese Academy of Sciences, Beijing, 100044 China; 2 CAS Center for Excellence in Life and Paleoenviroment, Beijing, 100044 China; 3 State Key Laboratory of Palaeobiology and Stratigraphy, Nanjing Institute of Geology and Palaeontology, Chinese Academy of Sciences, Nanjing, 210008 China; 4 College of Earth and Planetary Sciences, University of Chinese Academy of Sciences, Beijing, 100049 China; 5 Division of Paleontology, American Museum of Natural History, New York, 10024 USA

## Abstract

The earliest perissodactyls are represented by some basal equoid fossils from Euramerica near the Paleocene/Eocene boundary. Unequivocal early equoids have yet to be reported from the early Eocene of Asia, although other groups of early perissodactyls were indeed present in Asia. Here we report the earliest Eocene Asian equid, *Erihippus tingae* gen. et sp. nov., based on partial specimens initially assigned to the ceratomorph *Orientolophus hengdongensis*, from the Hengyang Basin of Hunan Province, China. The specimens previously assigned to ‘*Propachynolophus*’ *hengyangensis* from the same Lingcha fauna are split and now reassigned as an ancylopod *Protomoropus*? *hengyangensis* and a brontothere *Danjiangia lambdodon* sp. nov. The nearly simultaneous appearance of equids, ceratomorphs, ancylopods, and brontotheres in the Hengyang Basin suggests that the four main groups of perissodactyls diverged as early as, or no later than, the beginning of the Eocene (about 56 Ma), and displayed different dispersal scenarios during the early Eocene.

## Abstract (web summary)

Bin Bai et al. report a simultaneous appearance of equids, ceratomorphs, ancylopods, and brontotheres from the earliest Eocene Lingcha Fauna of the Hengyang Basin, China. This study suggests that the four main groups of perissodactyls diverged as early as the beginning of Eocene, and displayed different dispersal scenarios during the early Eocene.

## Introduction

The order Perissodactyla (odd-hoofed) consists of horses, tapirs, rhinos, and two extinct bizarre groups: chalicotheres bearing claws and brontotheres with pronounced horns in later forms^1^. The nearly simultaneous appearance of perissodactyls, artiodactyls, and primates on the Holarctic continents at the beginning of the Eocene about 56 Ma ago has obscured their places of origin and dispersal routes. Traditionally, perissodactyls were considered to have originated in North America or Asia (excluding India)^2,3^, but recent studies have favored the Indian subcontinent as the “Noah’s Ark” where perissodactyls originated^4^, and a hypothesis that the extinct South American ungulates were a sister group of perissodactyls should also be considered^5–7^. Nonetheless, non-Indian Asia remains as a competitive continent for the origin of perissodactyls based mainly on the controversial late Paleocene perissodactyls from Inner Mongolia^8^, as well as the perissodactyl-like *Radinskya* from the middle Paleocene of the Nanxiong Basin^9^.

The earliest perissodactyls have long been represented by the equoid ‘*Hyracotherium*’ from Europe and North America, which was historically considered to be the morphotype of early perissodactyls. However, North American ‘*Hyracotherium*’ was later split into several genera^10^, and the genus *Hyracotherium* restricted to its type species, *H*. *leporinum*, from Europe^11^. In consequence, the earliest equoids have subsequently been represented by *Cymbalophus cuniculus* and *Sifrhippus sandrae*, respectively, from PE I and Wa0 of Europe and North America near the Paleocene/Eocene boundary^12,13^. Unequivocal early Eocene equoids have yet to be reported from Asia since ‘*Hyracotherium*’ *gabuniai* from the Bumbanian strata of Mongolia was re-identified as belonging to the ancylopod *Protomoropus*^14–16^. *Gobihippus menneri* from the middle Eocene of Mongolia^17^ was considered to be either a palaeothere^18^ or a brontothere^19^.

The unequivocal earliest perissodactyls from Asia were the ceratomorph *Orientolophus hengdongensis* and the palaeothere ‘*Propachynolophus*’ *hengyangensis* from the upper Lingcha Fauna in the Hengyang Basin, Hunan Province^20–22^, as well as the lophialetid *Minchenoletes* from the Erlian Basin, Inner Mongolia of China^23^. The geologic section of the Lingcha Formation records the first Asian continental Carbon Isotope Excursion (CIE) in association with a mammalian fauna across the Paleocene/Eocene boundary^24^. The upper Lingcha Fauna bearing *O*. *hengdongensis, P. hengyangensis*, and other diverse mammals occurs stratigraphically from about 15 m above the Paleocene/Eocene boundary to the minimum carbon isotope value in the section of the Lingcha Formation (Supplementary Note 1)^25,26^; thus this fauna was considered slightly earlier than Wa0 of North America and PE 1 of Europe^27^, or nearly simultaneous with Wa0^28^. Some mammalian dispersal events have been inferred based on the age correlation^29,30^. Here we report an earliest Eocene equid from Asia based on two of the specimens originally assigned to *Orientolophus hengdongensis*, and re-identify the material of ‘*Propachynolophus*’ *hengyangensis* as a mixture of a chalicothere and a brontothere rather than a palaeothere. With these new perissodactyl taxa recognized, we further discuss potential dispersal routes of the early perissodactyls known from North America, Europe, and Asia.

## Results

### Systematic paleontology

Order Perissodactyla Owen, 1848

Suborder Hippomorpha Wood, 1937

Family Equidae Gray, 1821

*Erihippus tingae* gen. et sp. nov.

1993, *Orientolophus hengdongensis* (part) Ting, p. 202, fig. 1B-C.

### Etymology

*Eri* (Greek): early, at dawn; *hippos* (Greek): horse, a commonly used root in equid names; the specific name honors Prof. Su-Yin Ting, for her great contributions to the study of the early Eocene Lingcha Fauna in China.

### Holotype

IVPP V 5789.1, a left mandible with m1-3.

### Paratype

IVPP V 5790, a left maxilla with DP4-M2, which probably belongs to the same individual as V 5789.1. Their association is inferred from the fact that V 5789.1 and V 5790 have a similar slightly worn condition on upper and lower molars, the enamels of the lingual and occlusal surfaces are somewhat etched compared with the buccal side, and both of them are from the left side.

### Geological age

The specimens of *Erihippus tingae* were discovered from the Lingcha Formation, about 1 km southwest of Hetang village, Hengdong County, Hunan Province, China^20^. Earliest Eocene.

### Diagnosis

**Fig. 1:**
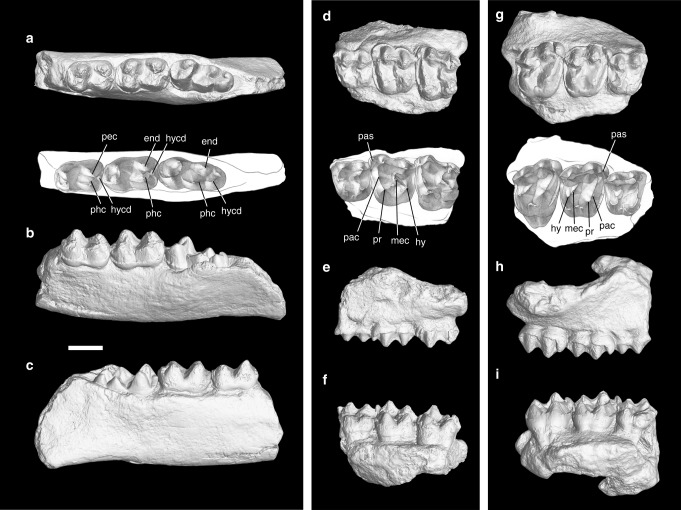
The earliest Eocene equid *Erihippus tingae* gen. et sp. nov. and ceratomorph *Orientolophus hengdongensis* Ting, 1993 from the Lingcha Fauna of Hunan Province, China. **a**–**f** *Erihippus tingae*, **a**–**c** left mandible with m1-3 of IVPP V 5789.1 in occlusal (**a**), buccal and slightly occlusal (**b**), and lingual views (**c**); **d**–**f**, left maxilla with DP4-M2 of IVPP V 5790 in occlusal (**d**), buccal (**e**), and lingual views (**f**); **g**–**i** *Orientolophus hengdongensis*, right maxilla with DP4-M2 of IVPP V 5789 in occlusal (**g**), buccal (**h**), and lingual views (**i**). Abbreviations: end entoconid; hy hypocone; hycd hypoconulid; mec metaconule; pac paraconule; pas parastyle; pec postentocristid; phc posthypocristid; pr protocone. Scale: 5 mm

Relatively high lophodonty of M1-2 with small paraconules and metaconules; Paraconules situated in the midpoint of the protolophs; metalophs terminate with metacone folds at the mesiolingual side of the metacone; entoconid of m3 completely separated from the hypoconid (Fig. 1a–f). Differs from both *Sifrhippus* and *Cymbalophus* in the lack of lingual cingula on the upper molars, and in a more convex rib on the buccal surface of the metacone on upper molars, a metaconid not twinned but with a weak metaconid buttress on lower molars, and a hypoconulid of m3 relatively larger with a small cuspid distolingually placed (Supplementary Figs. 1, 3). Further differs from *Sifrhippus* by the lack of a crest on the lingual side of the trigonid on m1-2, and by a cristid obliqua more buccally extended on lower molars, and a posthypocristid joining the hypoconulid on m2. Further differs from *Cymbalophus* in more plane lingual surfaces of the molar paracones, more continuous buccal cingula on M1-2, the hypoconulid of m3 separated from the posthypocristid by a notch, and the lack of a rather weak “hypolophid” connecting the hypoconid and entoconid on m3 (Supplementary Fig. 1).

### Comparative descriptions

Ting^20^ reported and described *Orientolophus hengdongensis* from the Lingcha Formation of the Hengyang Basin, China. The phylogenetic position of *Orientolophus* has been disputed either as a basal ceratomorph^3,31^ or a hippomorph^16,32^. The material originally referred to *O*. *hengdongensis* consists of three specimens: a right maxilla with DP4-M2 (IVPP V 5789), a left mandible with m1-3 (IVPP V 5789.1), and a left maxilla with DP4-M2 (IVPP V 5790) (Fig. 1). However, the left mandible (IVPP V 5789.1) and the left maxilla (IVPP V 5790) differ from *Orientolophus* and are assigned to a new equid genus *Erihippus*. Only the right maxilla (IVPP V 5789) represents *Orientolophus*.

The following comparative description is mainly based on *Erihippus tingae* in comparison with early equid *Sifrhippus sandrae* from Wa0 of North America^10,13^, *Cymbalophus cuniculus* from PE I of Europe^11,12,33^, and the early Eocene tapiromorphs *Orientolophus*, *Chowliia*^32^, and *Cardiolophus*^34^ (Supplementary Fig. 1, Supplementary Note 2).

IVPP V 5790

DP4: The tooth is identified as deciduous, because fragmentary enamel beneath the tooth probably belongs to an unerupted P4, and the enamel of the crown is slightly lighter in color (Fig. 1d–f; Supplementary Fig. 1a). However, no enamel is discernible beneath the DP4 of *Orientolophus* (IVPP V 5789). The tooth is moderately worn, roughly quadrate in outline as in *Sifrhippus*, while that of *Orientolophus* is slightly wider than long. The protocone and the hypocone are partially broken. The paracone and metacone are somewhat mesiodistally compressed, conical, and bear equally-developed ribs on buccal and lingual sides as in *Orientolophus*, in contrast to the less convex rib on the buccal side of the metacone in *Sifrhippus*. The centrocrista between the paracone and metacone is straight and deeply notched without a mesostyle (Fig. 1e). The postmetacrista is mainly distally directed. The parastyle is small, and mesiobuccal to the paracone. The protocone is situated at the level of the paracone, extending a weak preprotocrista to a distinct paraconule, which is more mesially placed. The metaconule is also distinguishable, and placed mesiobuccal to the hypocone. The distinct paraconule and metaconule are also present in *Sifrhippus* and other early equids, whereas those of *Orientolophus* are not discernible or weak as in early tapiromorphs. The premetaconule crista extends toward the mesiolingual base of the metacone and slightly curves upward as in *Sifrhippus*, whereas the low metaloph ends at the mesiolingual base of the metacone in *Orientolophus*. Cingula are absent on the lingual side, but weakly developed on the mesial, buccal, and distal sides. In contrast, a distinct cusp is present at the base of the central valley in *Sifrhippus* and *Orientolophus*^35^.

M1: This tooth is distinctly larger than DP4 (Fig. 1d–f; Supplementary Figs. 1a, 3, Supplementary Table 1). It is slightly worn and moderately lophodont. The outline of the crown is roughly rectangular with the width greater than the length. The buccal side of the paracone bears a distinct, somewhat mesiobuccally compressed rib, whereas the lingual side is generally flat as in *Sifrhippus*. In *Cymbalophus* and *Orientolophus*, the lingual side of the paracone is convex. The metacone is more lingually placed than the paracone, with a mesiobuccally compressed rib on the buccal side and inflated surface on the lingual side as in *Orientolophus*, whereas the buccal metacone ribs of other compared taxa are much weaker or nearly flat. As in *Sifrhippus* and *Cymbalophus*, the paracone and metacone are relatively more widely separated than those of *Orientolophus* (Fig. 1e, h). The separation can be inferred by a wider groove on the buccal side between the paracone and the metacone. The centrocrista is straight without a mesostyle. The preparacrista is mesially and slightly lingually extended, whereas the postmetacrista is distally extended. The parastyle is considerably smaller than the paracone and mesial to the latter; although, the parastyle is relatively larger than that of DP4. The relatively small parastyle resembles those of *Sifrhippus* and *Cymbalophus*, while the parastyle of *Orientolophus* is relatively large as in other early tapiromorphs (Fig. 1d, g). The protocone is situated at the level of the paracone, whereas the hypocone is slightly more distally placed relative to the level of the metacone. The paraconule is distinct, mesiobuccal to the protocone, and situated roughly in the middle of the protoloph as in *Sifrhippus* and *Cymbalophus*, while the paraconules of *Orientolophus* and other early tapiromorphs are relatively small and placed close to the protocone (Fig. 1d, g). The protoloph, which is composed of the preprotocrista and preparaconule crista, is generally lophodont and extends to the preparacrista, while that of *Orientolophus* is slightly higher and more prominent as in early tapiromorphs. The hypocone is slightly buccal to the protocone, as shown by the slightly buccally oblique lingual border of the crown. The metaconule is distinct and mesiobuccal to the hypocone, but smaller than the paraconule as in *Sifrhippus* and *Cymbalophus*, whereas the metaconules of *Orientolophus* and other early tapiromorphs are relatively more reduced. The premetaconule crista extends to the mesiolingual base of the metacone, ending up with a short metacone fold as in *Sifrhippus*, *Cymbalophus*, and *Chowliia*, while the metalophs of *Orientolophus*, *Cardiolophus*, and *Homogalax* end at the base of the metacones without metacone folds (Fig. 1d, g). Cingula are similar to those in DP4, but are more distinct. The lack of a lingual cingulum on M1 in *Erihippus* is different from the lingual cingulum that is complete or only interrupted at the hypocone in *Cymbalophus* and *Sifrhippus*. In contrast, the lingual cingulum of M1 is present at the end of the central valley in *Orientolophus* as in other early tapiromorphs.

M2: This tooth is slightly worn, and the parastyle and the apices of the metacone, protocone, and hypocone are broken (Fig. 1d–f; Supplementary Figs. 1a, 3, Supplementary Table 1). The morphology of M2 is similar to that of M1, except that the metacone has a less mesiodistally compressed rib on the buccal side.

IVPP V 5789.1

m1: This tooth is rectangular in outline with the talonid slightly wider than the trigonid (Fig. 1a–c; Supplementary Figs. 1e, 3, Supplementary Table 1). The protoconid and the metaconid are conical, closely placed, and with the latter slightly more distally situated. The protolophid is deeply notched. The paralophid extends from the protoconid mesially to the mesiobuccal corner, and then lingually to the mesial base of the metaconid. The longitudinally orientated paralophid of m1 in *Erihippus* is similar to that of *Cymbalophus* and early tapiromorphs, whereas that of *Sifrhippus* is slightly more lingually extended (Supplementary Fig. 1e-g). The metaconid is not twinned, but a weak metaconid buttress is discernible on the distobuccal side of the metaconid. In contrast, the metaconids in other compared taxa are distinctly twinned. The hypoconid and entoconid are conical, and the latter is slightly more distally placed. The posthypocristid and the postentocristid form a deeply notched postcristid and join in the middle where a large hypoconulid is situated distally. The notched, complete postcristid, which is composed of posthypocristid and postentocristid, has been considered to be the primitive state in postcristid/hypolophid complex^36^. The postcristid of m1 in *Erihippus* resembles those of *Sifrhippus* and *Cymbalophus*, distinctly contrasting with unnotched, nearly lophodont hypolophids in early tapiromorphs^12,32^. The cristid obliqua extends from the hypoconid mesially to a point buccal to the midpoint of the protolophid as in *Cymbalophus* and early tapiromorphs, and terminates in a moderately high position. In contrast, the cristid obliqua in *Sifrhippus* is slightly more lingually extended. A weak mesial cingulum is continuous on the buccal side of the protoconid, and a distal cingulum is present. The lingual side of the crown and the buccal side of the hypoconid lack the cingula.

m2: This tooth is similar to m1, but m2 is larger with the talonid slightly narrower than the trigonid (Fig. 1a–c; Supplementary Figs. 1e, 3, Supplementary Table 1). The main difference between m2 and m1 is that the posthypocristid of m2 is distolingually extended, joining the large hypoconulid; whereas, the postentocristid is absent. The entoconid is separated from the hypoconid and encircled by a deep, narrow groove on the buccal side. The postcristid on m2 with its broken postentocristid is more derived than that of m1. However, the postcristids of m2 in *Cymbalophus* remain as complete as those of m1, whereas those of *Sifrhippus* are either complete or with the broken postentocristid. In contrast, the hypolophids of m2 in early tapiromorphs are nearly unnotched and lophodont.

m3: This tooth is not completely erupted and the crown is unworn (Fig. 1a–c; Supplementary Figure 1e, 3). The trigonid is similar to those of m1 and m2 with a deeply notched protolophid, but the metaconid buttress is more reduced. The cristid obliqua extends from the hypoconid mesially and turns slightly lingually at the mesial end, whereas the posthypocristid extends distolingually and is separated from the hypoconulid by a deep notch. In contrast, the posthypocristids of m3 in early tapiromorphs are completely absent. The entoconid is roughly conical, with a flat, triangular mesial surface, and is completely isolated from the hypoconid and hypoconulid as in *Sifrhippus* and most *Cymbalophus*, which usually bears a much weak hypolophid. In contrast, the hypolophids of m3 in early tapiromorphs are lophodont or shallowly notched. The hypoconulid is large, buccally situated, and bears mesial and distal ridges. The mesial ridge joins the posthypocristid but is separated from the latter by a deep notch as in *Sifrhippus* and *Cymbalophus*. A smaller cusp is situated distolingual to the hypoconulid, and separated from the latter by a moderately deep valley. A small cuspid lingual to the hypoconulid is also present in *Sifrhippus* and *Cymbalophus*, but is relatively more mesially placed. The cingulum is weak and discontinuous on the mesiobuccal side.

To sum up, we propose a new genus and species, *Erihippus tingae*, for IVPP V 5789.1 and V 5790, which represents a basal Asian equid and is similar to nearly contemporaneous *Sifrhippus* and *Cymbalophus* in having a small parastyle, distinct paraconule and metaconule with the former placed in the middle of the protoloph, deeply notched postcristids on m1-2, the hypoconid and the entoconid of m3 completely separated, and the posthypocristid of m3 consistently present and joining the hypoconulid. These features are relatively stable and rarely susceptible to the intraspecific variation (Supplementary Fig. 1, Supplementary Note 2)^36,37^. *Orientolophus hengdongensis* should only be represented by the right maxilla with DP4-M2 (IVPP V 5789).

In contrast, the M1-2 of *Orientolophus* (IVPP V 5789) is similar to that of *Chowliia*^32^ and *Cardiolophus*^34^ mainly in having a relatively large parastyle, relatively more lophodont protoloph and metaloph, and the paraconule placed close to the protocone.

Suborder Tapiromorpha Haeckel, 1866

Infraorder Ceratomorpha Wood, 1937

Family inc. sed.

*Orientolophus hengdongensis* Ting, 1993

### Lectotype

IVPP V 5789, a right maxilla with DP4-M2.

### Geological age

Same as *Erihippus tingae*.

### Emended Diagnosis

Differs from *Cymbalophus*, *Sifrhippus*, and *Erihippus* in more lophodont teeth with relatively smaller paraconules and metaconules, a larger parastyle on M1-2, the protoloph of M1-2 extending toward the parastyle, the paraconule closer to the protocone than to the paracone, the lingual cingula of M1-2 restricted in the opening of the central valley, and in the lack of the metacone fold. Further differs from *Cymbalophus* in the squarer outline of M1, a parastyle mesial or slightly buccal to the paracone, the metacone rib more convex on the buccal side on M1-2, and the complete cingulum on the buccal side of the paracone on M1-2. Further differs from *Erihippus* in the paracone of M1-2 less mesiodistally compressed on the buccal side and more swollen on the lingual side (Fig. 1g–i; Supplementary Figs. 1, 3).

Suborder Tapiromorpha Haeckel, 1866

Infraorder Ancylopoda Cope, 1889

Family inc. sed.

*Protomoropus* Hooker and Dashzeveg, 2004

*Protomoropus*? *hengyangensis* (Young, 1944)

1944, *Propalaeotherium hengyangensis* Young, p. 1, fig. 1.

1965, *Propalaeotherium hengyangensis*, Savage, Russell & Louis, p. 72.

1979, *Propachynolophus hengyangensis* (part) Li et al., p. 74, pl. 1, fig. 1c.

2003, *Propachynolophus hengyangensis* (part) Ting et al., p. 524, fig. 2.

### Holotype

IVPP V 214, a left mandible with m3.

### Referred specimens

V 7453, a left m3.

### Geological age

V 214 was discovered from field locality no. 76003, about 200 m south of Lingcha, Hengyang Basin^21,22^. V 7453 was found from the same locality where *Erihippus* and *Orientolophus* were discovered^26^. Earliest Eocene.

### Diagnosis

**Fig. 2:**
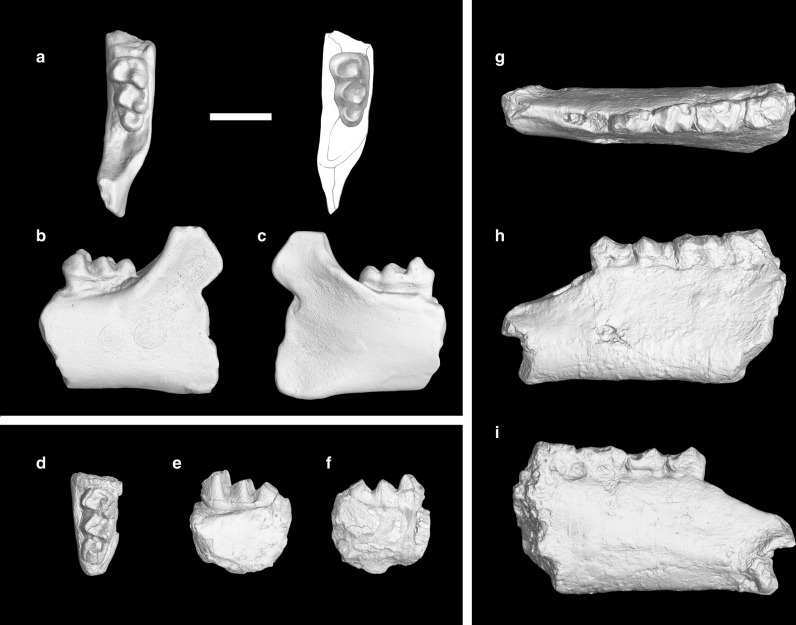
The earliest Eocene ancylopod *Protomoropus*? *hengyangensis* (Young, 1944) and brontothere *Danjiangia lambdodon* sp. nov. from the Lingcha Fauna of Hunan Province, China. **a**–**f**, *Protomoropus*? *hengyangensis*, **a**–**c**, left mandible with m3 of IVPP V 214 (cast) in occlusal (**a**), buccal (**b**), and lingual views (**c**); (**d**–**f**), left m3 of IVPP V 7453 in occlusal (**d**), buccal (**e**), and lingual views (**f**). **g**–**i** *Danjiangia lambdodon*, left mandible with p3-m2 of IVPP V 5349 in occlusal (**g**), buccal (**h**), and lingual views (**i**). Scale: 1 cm

Differs from *Protomoropus gabuniai* in a deeply notched hypolophid on V 214, and in the lack of a cingulum on the buccal side of the hypoconulid lobe; Differs from *Pappomoropus taishanensis* in a more lingually extended buccal branch of the paralophid, a more lophodont hypolophid on V 7453, and a relatively larger and basined hypoconulid lobe (Fig. 2a–f; Supplementary Figs. 2a-f, 3).

### Comparative descriptions

Young^21^ originally described a fragmentary left mandible with m3 (IVPP V 214) as ‘*Propalaeotherium*’ *hengyangensis*, now *Protomoropus*? *hengyangensis*, from the Hengyang Basin, Hunan Province, China. At the time, this specimen represented the first determinable fossil mammal from South China^38^. questioned its affinity with *Propalaeotherium* but without confident evidence because only a single m3 was then known^22^. subsequently reported an additional left mandible with p3-m2 from the same locality and re-identified the species as ‘*Propachynolophus*’ based on its small size and relatively low degree of molarized premolars (p3 with indistinct metaconid). Ting (1995)^26^ reported an additional specimen of m3 (IVPP V 7453) from the same basin. She further suggested a close relationship of ‘*P*.’ *hengyangensis* with chalicotheres, which is represented by the late early Eocene *Litolophus gobiensis* known from Inner Mongolia of China^39–42^, based mainly on the cristid obliqua of the lower molars connecting to the protolophid more lingually than that of ceratomorphs and more buccally than that of hippomorphs, and the hypoconulid of m3 forming an enclosed, circular basin rather than a main ridge with wide valleys buccal and lingual to it as in *Propachynolophus*. More recent study, however, suggested that ‘*P*.’ *hengyangensis* resembles *Danjiangia* based on p3-m2 morphology^32^. Both *Danjiangia* and ‘*Propalaeotherium*’ *sinense* have striking similarities to *Lambdotherium*, a basal brontothere^43^. However, we suggest that the three specimens that Ting referred to ‘*P*.’ *hengyangensis* probably represent at least two different groups of perissodactyls. IVPP V 214 and V 7453 are similar to the early ancylopods *Protomoropus*^15^ or *Pappomoropus*^32^, whereas V 5349 can be assigned to *Danjiangia*^44^. In the following comparative description, we compare the Hengyang specimens with the palaeothere *Propachynolophus maldani*, the ancylopods *Protomoropus* and *Pappomoropus*, and the brontotheres *Danjiangia* and *Lambdotherium*.

The holotype m3 of *Protomoropus*? *hengyangensis* (IVPP V 214) is missing, and only a cast is currently available. m3: The following description was based on the cast of IVPP V 214 with reference to Young^21^ (Fig. 2a–c; Supplementary Fig. 2a-c, Supplementary Table 1). The tooth is unworn and tapers distally. The paralophid extends mesiolingually from the protoconid, and then extends lingually to the mesial base of the metaconid. In contrast, the buccal branch of m3 paralophids in *Danjiangia* and *Lambdotherium* extend more lingually (Supplementary Fig. 2j), while that of *Pappomoropus* is nearly longitudinally orientated. The metaconid is slightly distally placed relative to the protoconid, and a distinct twinned metaconid (‘metastylid’) is situated distolingual to the metaconid. The protolophid is nearly transverse and moderately notched. The talonid is narrower than the trigonid. The cristid obliqua extends mesiolingually from the hypoconid to the protolophid slightly lingual to the midpoint, and ends in a relatively low position as in *Protomoropus* and *Pappomoropus*. In contrast, the cristids obliquae of m3 in *Propachynolophus*, *Danjiangia*, and *Lambdotherium* are high and more lingually extended (Supplementary Fig. 2j-k). The entoconid is lower than the hypoconid and more distally placed. The hypolophid is oblique and relatively deeply notched. On the lingual border of the talonid are a number of small, fine wrinkles as described by Young^21^. The hypoconulid is enlarged into a third lobe, which forms an enclosed, circular basin and is distinctly smaller than the talonid. The buccal ridge of the hypoconulid lobe joins the middle of the hypolophid, whereas the lingual one connects to the distal base of the entoconid. The circular basin of the m3 hypoconulid is also discernible in *Protomoropus*, whereas the hypoconulid lobes of *Propachynolophus*, *Danjiangia*, and *Lambdotherium* are relatively narrow and elongated with a more lingually oriented buccal ridge and a prominent cuspid representing the hypoconulid. The hypoconulid of m3 in *Pappomoropus* is partially in its crypt, but it is clear that the hypoconulid lobe is much lower than the talonid and does not form a basin, with a buccal ridge extending from a distinct cuspid (hypoconulid) to the midpoint of the hypolophid. Young^21^ also mentioned that a distinct cingulum is present on the buccal side and extends to the mesial side.

Ting^26^ described a second specimen of m3 (IVPP V 7453) in detail (Fig. 2d–f; Supplementary Fig. 2d-f, Supplementary Table 1), thus it is not necessary to replicate here. Although V 7453 and the holotype are similar in general morphology, V 7453 is different from V 214 in being slightly longer and narrower, and in having a higher, more acute protolophid, hypolophid, and cristid obliqua as determined by Ting^26^. V 7453 further differs from V 214 in being less distally tapering, and in having a relatively wider hypoconulid lobe with its distal side mesially inclined as in *Protomoropus*. Whether these differences are attributed to intraspecific variation or suggest two different groups is uncertain, pending the discoveries of more complete material. As a result, considering the similarities in size and general morphologies of m3 between V 214, 7453 and *Protomoropus*, we tentatively assigned the two specimens to the same species, *Protomoropus*? *hengyangensis*.

Suborder Titanotheriomorpha Hooker, 1989

Family Brontotheriidae Marsh, 1873

*Danjiangia* Wang, 1995

*Danjiangia lambdodon* sp. nov.

1979, *Propachynolophus hengyangensis* (part) Li et al., p. 74, pl. 1, fig. 1a-b. 

2003, *Propachynolophus hengyangensis* (part) Ting et al., p. 524.

### Lambda (Greek)

 eleventh letter of the Greek alphabet, indicating the V-shaped talonid on lower molars; *odon* (Greek): tooth, a commonly used suffix in mammalian names.

### Holotype

IVPP V 5349, a left mandible with p3-m2.

### Geological age

V 5349 were discovered from field locality no. 76003, where V 214 was also found, about 200 m south of Lingcha, Hengyang Basin^21,22^. Earliest Eocene.

### Diagnosis

Differs from *Danjiangia pingi*^44^ and *Lambdotherium*^45^ in the more distinct hypolophid and entoconid on p3-4, a more oblique hypolophid on m1-2, and a relatively deeper horizontal ramus of the mandible (Fig. 2g–i; Supplementary Figs. 2g-k). Further differs from *Danjiangia pingi* in the cristids obliquae of the lower molars connecting to protolophids buccal to ‘metastylids’.

### Comparative descriptions

A left mandible (V 5349) preserves heavily worn p3-m2 and an alveolus for p2 (Fig. 2g–i; Supplementary Fig. 2g-i, Supplementary Table 1). The alveolar border descends considerably mesial to p3, and the ventral border of the horizontal ramus is nearly straight. The preserved horizontal ramus is relatively deeper than those of *Danjiangia*, *Lambdotherium*, *Propachynolophus*, and *Pappomoropus* (Supplementary Fig. 2g-k). The alveolus for p2 is composed of two parts: the mesial one is oval, narrower, whereas the distal one is rounded, wider. The length of p2, as inferred from the alveolus, is about 6.18 mm. Mesial to the alveolus for p2 is a diastema with a broken mesial end, indicating a long diastema between p1 and p2 as in *Danjiangia* and *Pappomoropus*, or (less probably) the p1 is absent as in *Lambdotherium*. A mental foramen is present below p3.

p3: This tooth is moderately worn, and the crown is roughly oval with a talonid wider than the trigonid (Fig. 2g–i; Supplementary Fig. 2g-i). The protoconid (or fused protoconid and metaconid) is conical and forms the main cusp of the crown as in *Danjiangia* and *Lambdotherium*, linked mesially by a short paracristid to a relatively high paraconid. In contrast, the metaconid of p3 is large and as high as the protoconid in *Propachynolophus* and *Pappomoropus*. A small cuspule is probably present distolingual to the protoconid as in *Danjiangia* and *Lambdotherium*. The hypoconid is distinct, with a cristid obliqua extending mesiolingually to the distolingual side of the protoconid in a relatively high position. A weak, low hypolophid extends lingually from the hypoconid to a rudimentary entoconid as in *Lambdotherium*, while the hypolophids in the other compared taxa are absent or very short. A weak cingulum is present on the mesial part of the buccal side.

p4: This tooth is moderately worn and roughly rectangular in outline with the talonid slightly wider than the trigonid (Fig. 2g–i; Supplementary Fig. 2g-i). The paralophid extends mesially and slightly lingually from the protoconid to a low paraconid. The metaconid is as large as the protoconid, and slightly more distally placed. The metaconid is twinned as in other compared taxa, although the separation of the twinned metaconid has been obliterated by the wear. The cristid obliqua extends mesiolingually from the hypoconid to the twinned metaconid in a relatively high position. The hypolophid is a narrow ridge extending from the hypoconid to a low entoconid, which is more distinct than that of p3. In contrast, the hypolophids of p4 are relatively low with inconspicuous entoconids in the other compared taxa. A weak cingulum is present at the mesiobuccal corner of the crown.

m1-2: These teeth are heavily worn, and the morphology of m2 is nearly obliterated mainly due to the breakage (Fig. 2g–i; Supplementary Figs. 2g-i, 3). The metaconid of m1 is broken off. The crown of m1 is rectangular, with the talonid slightly wider than the trigonid. The trigonid is wider than long with a somewhat lingually extended paralophid. The cristid obliqua extends mesiolingually from the hypoconid to the buccal side of the twinned metaconid as in *Lambdotherium*. In contrast, the cristid obliqua of m1-2 in *Danjiangia pingi* extends to the ‘metastylid’, and those of early chalicotheres extend to a point more buccal than the twinned metaconid in a relatively lower position. The hypolophid is distinctly oblique, extending distolingually from the hypoconid to a prominent entoconid. The hypolophid of m1-2 in *D*. *lambdodon* is more oblique than those of other compared taxa. A weak cingulum is present at the buccal part of the mesial border.

Considering the similarities in size and general morphologies of p3-m2 between V 5349 and *Danjiangia pingi* as discussed above, we assigned the specimen to *Danjiangia*. The new species *D*. *lambdodon* is erected mainly based on its more molariform p3-4 than in *D*. *pingi*.

### Results of the phylogenetic analysis

**Fig. 3:**
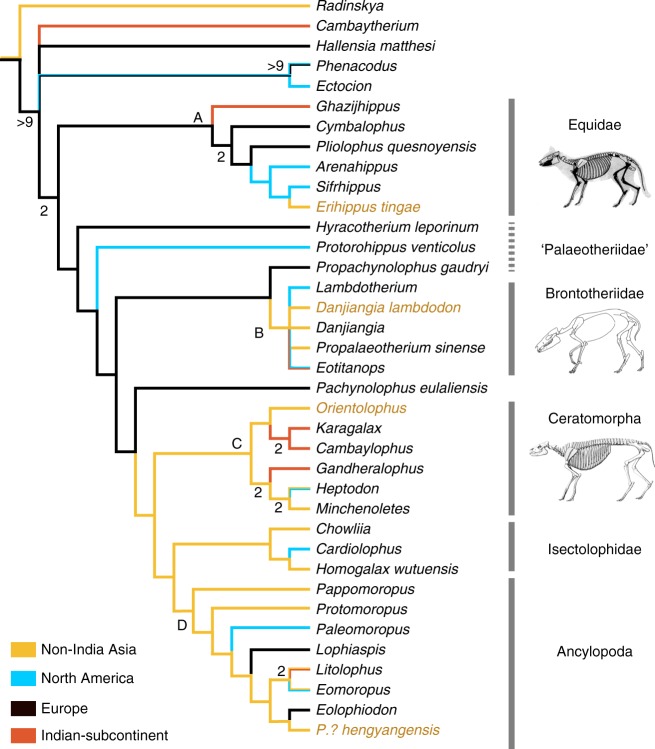
The phylogenetic relationships of early perissodactyls and ancestral reconstruction of the geographic distribution. The tree is the strict consensus of 63 equally most parsimonious trees. The data matrix consists of 37 taxa and 70 characters. The Bremer Support that is greater than 1 was shown at the node. Four species from the earliest Eocene deposits in the Hengyang Basin, China are marked in yellowish brown. The geographic distribution was reconstructed using parsimonious criterion in Mesquite 3.31^61^. The skeletal reconstructions are *Sifrhippus* (with permission from Gingerich^13^), *Lambdotherium*^62^ (U.S. Geological Survey), and *Heptodon*^63^ (Museum of Comparative Zoology, Harvard University), respectively, from the top to the bottom

A cladistic analysis with parsimony criteria results in 63 equally most parsimonious trees (Supplementary Note 3). The strict consensus tree (Fig. 3; Supplementary Fig. 4, Supplementary Table 2) shows that *Sifrhippus* and *Erihippus* form a sister group, and *Arenahippus* is the sister group to *Sifrhippus*-*Erihippus* clade. Considering *Arenahippus* has a very similar morphology to *Sifrhippus* and appeared slightly above the horizon bearing *Sifrhippus*^28^, a more basal position of *Arenahippus* is not unexpected. European *Cymbalophus*^12^ and *Pliolophus quesnoyensis*^37^ form successive sister-taxa lineages to North American and Asian equids. Indian *Ghazijhippus*^46^ is placed at the most basal position of equids. Equidae is situated at the most basal position of perissodactyls. *Danjiangia lambdodon* is allied with Brontotheriidae, but the relationships within brontotheres remain polytomous. However, another controversial taxon from China, ‘*Propalaeotherium*’ *sinense*^47^, is also placed in the Brontotheriidae clade. Brontotheres are placed at a relatively more derived position than equids among perissodactyls mainly because the brontotheres have strong mesostyles on upper molars and lophodont hypolophids on lower molars (node 56 in Supplementary Fig. 4). *Orientolophus* is in the clade Ceratomorpha, forming a sister group with *Karagalax*-*Cambaylophus*^48,49^ from India. Isectolophidae (*Chowliia*, *Cardiolophus*, and *Homogalax*) is closer to Ancylopoda than to Ceratomorpha^50^. *Protomoropus*? *hengyangensis* is placed in Ancylopoda; however, its close relationship with European *Eolophiodon*^51^ is open for discussion and can be attributed to only m3 preserved in *P*.? *hengyangensis*. The strict consensus tree also supports Ceratomorpha and Isectolophidae-Ancylopoda forming a monophyletic Tapiromorpha. However, in contrast to the traditional viewpoints, palaeotheres are not sister group to equids, but closer to other non-equid perissodactyls. *Propachynolophus gaudryi* forms a sister group with brontotheres, whereas *Pachynolophus eulaliensis* is a sister group to Tapiromorpha. *Hallensia*, which was considered either to be a condylarth^52^ or an equoid^53^, is situated outside of Perissodactyla. Phylogenetic analyses with postcranial characters suggest that *Hallensia* is either the sister group to perissodactyls^54^ or placed within equoids^10,31^.

## Discussion

The phylogenetic positions of *Erihippus*, *Orientolophus*, *Danjiangia lambdodon*, and *Protomoropus*? *hengyangensis* in Fig. 3 are consistent with morphological comparisons. The simultaneous appearance of four main groups of Perissodactyla in the Lingcha Formation, Hengyang Basin, suggests that the divergence of Equidae, Brontotheriidae, Ceratomorpha, and Ancylopoda occurred near, or no later than, the Paleocene/Eocene boundary, about 56 Ma, specifically from about 15 m above the Paleocene/Eocene boundary to the minimum carbon isotope value during the Paleocene-Eocene Thermal Maximum (PETM)^24^. The split between equids and ceratomorphs at about 56 Ma is earlier than the estimated divergence time (mean 48.88 Ma) based on the mitochondrial genome^5^, but is slightly later than previous estimates based on the controversial fossil record (58 Ma)^55^. The divergence of four perissodactyl groups near the Paleocene/Eocene boundary suggests that the origin of perissodactyls should took place within the Paleocene^1,5^.

**Fig. 4:**
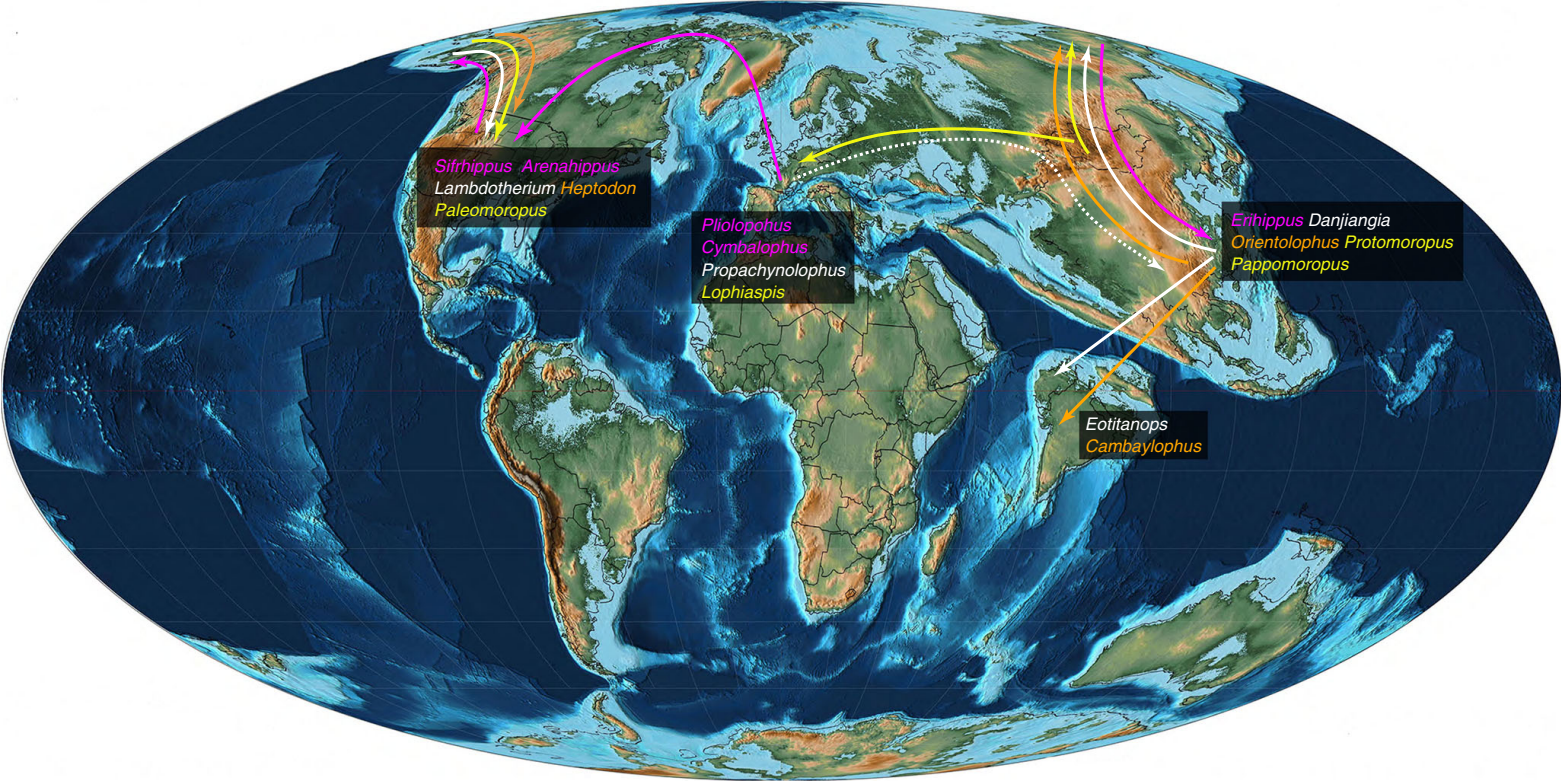
Paleogeographic map of the world during PETM^64^, and the dispersal routes of early perissodactyls based on the ancestral reconstruction of the geographic distribution using parsimonious criterion by Mesquite 3.31^61^ (refer to Fig. 3). The magenta lines and taxa show that equids originated from Europe and dispersed westward to North America and then to Asia. The solid white and orange lines and taxa show that brontotheres and ceratomorphs, respectively, originated from non-India Asia and dispersed to North America and the Indian-subcontinent; the dotted white line suggests that Asian brontotheres probably derived from palaeotheres and dispersed from Europe. The solid yellow lines and taxa show that ancylopods originated from Asia and dispersed eastward to North America and westward to Europe. The Paleogeographic map is reproduced with the permission from Scotese^64^

Based on ancestral reconstructions of the geographic distribution using the parsimonious criterion, we suggest that equids originated in Europe (node A of Fig. 3). One clade dispersed to the Indian-subcontinent giving rise to *Ghazijhippus*^46^, probably along the northern margin of the Neotethys^56^, while the other clade gave rise to European *Cymbalophus* and *Pliolophus quesnoyensis*. The European equids dispersed to North America via the Greenland land bridge giving rise to *Arenahippus* and *Sifrhippus*, which immigrated to Asia via the Bering Strait giving rise to *Erihippus* during the PETM (Fig. 4). The relatively basal positions of *Cymbalophus* and *Pliolophus quesnoyensis* favor the hypothesis of PE I as latest Paleocene in age^57^, slightly earlier than the Asian Lingcha Fauna and North American Wa0. It is noteworthy that although few postcrania of early Eocene equids have been found from Europe, the early middle Eocene horses from Messel are even more primitive than the considerably older hyracotheres from early Eocene of North America^58^. The lower part of the upper Ghazij Formation bearing *Ghazijhippus* has been considered to be early Eocene and older than the North American Wasatchian-Bridgerian mammalian faunal transition^46^, suggesting that *Ghazijhippus* appeared later than other early equids.

The analysis further suggests that Brontotheriidae likely originated from Asia (excluding the Indian-subcontinent) (node B of Fig. 3), and then dispersed to North America in the late Wasatchian and to the Indian-subcontinent in the late early Eocene (Fig. 4). Furthermore, brontotheres probably derived from some palaeothere groups, such as *Propachynolophus*, and dispersed from Europe (Fig. 3). Although only one relatively derived species of *Propachynolophus*, *P*. *gaudryi*, was included in the matrix, the genus *Propachynolophus* appeared as early as MP 8-9 as represented by *P*. *levei*^11^, which could have dispersed from Europe to Asia. However, the species previously assigned to *Propachynolophus* remain controversial and probably represent different evolutionary lineages^59^.

Ceratomorpha originated in non-Indian Asia (node C of Fig. 3). During the early Eocene, ceratomorphs dispersed to the Indian-subcontinent twice as represented by the *Karagalax*-*Cambaylophus* clade and *Gandheralophus*, and dispersed to North America as represented by *Heptodon* (Fig. 4). Ancylopoda (node D of Fig. 3) also originated in non-Indian Asia as *Pappomoropus* and *Protomoropus* representing the basal groups. Ancylopoda dispersed to North America eastward and to Europe westward, giving rise of *Paleomoropus* and *Lophiaspis*, respectively. Further, palaeothere *Pachynolophus eulaliensis* is the sister group to Tapiromorpha, suggesting that the latter probably also derived from some palaeotheres and dispersed from Europe as brontotheres did.

## Methods

### Imaging and figure

Optical images were taken using a Nikon D3X digital camera. Micro-CT was utilized in order to enhance observation of the morphology. Scanning was carried out using 225 kV micro-computerized tomography (developed by the Institute of High Energy Physics, Chinese Academy of Sciences (CAS)) at the Key Laboratory of Vertebrate Evolution and Human Origins, CAS. The beam energy, the flux, and the resolution per pixel for each specimen are as follows: IVPP V 5789.1, 130 kV, 120 μA, 25.09 μm; V 5790, 120 kV, 120 μA, 13.33 μm; V 5789, 130 kV, 120  μA, 14.90 μm; V 214, 100 kV, 100 μA, 21.96 μm; V 5349, 130 kV, 100 μA, 31.37 μm; and V 7453, 110 kV, 120 μA, 10.98 μm. A 360° rotation with a step size of 0.5° and an unfiltered aluminium reflection target were used. A total of 720 transmission images were reconstructed in a 2048 × 2048 matrix of 1536 slices in each scan using a two-dimensional reconstruction software developed by the Institute of High Energy Physics and Institute of Automation, CAS. The three-dimensional reconstructions were performed using software VG Studio 2.1.

### Phylogenetic analysis

We performed phylogenetic analysis using PAUP 4.0a157 with a parsimony criterion^60^. All characters are unordered and equally weighted. The number of replications of random stepwise addition is 1000 with 10 trees held at each step. Tree-bisection-reconnection (TBR) is used and set up with reconnection limit equal to eight. “MulTrees” option is in effect. Branches collapsed (creating polytomies) if minimum branch length is zero. The analysis results in 63 most parsimonious tress. The tree length of the strict consensus tree is 341; the consistency index (CI) is 0.3607; the homoplasy index (HI) is 0.6393; the retention index (RI) is 0.6228; the rescaled consistency index (RC) is 0.2247.

### Data availability

All specimens (IVPP V 5790, V 5789.1, V 5789, V 5349, and V 7453) and the cast (IVPP V 214) are deposited at the Institute of Vertebrate Paleontology and Paleoanthropology, Chinese Academy of Sciences, Beijing, China. Supporting data (character list and data matrix) for the phylogenetic analyses for this study are provided in Supplementary Information. The data matrix was deposited in Morphobank (project 3210).

## Electronic supplementary material

Supplementary Information
