# Supplementary material for: The divergence and dispersal of early perissodactyls as evidenced by early Eocene equids from Asia
Source: Commun Biol. 2018 Aug 15;1:115. doi: 10.1038/s42003-018-0116-5 (PMC6123789; doi:10.1038/s42003-018-0116-5)
Supplement: Supplementary file 1 — Supplementary Information [file 42003_2018_116_MOESM1_ESM.pdf]

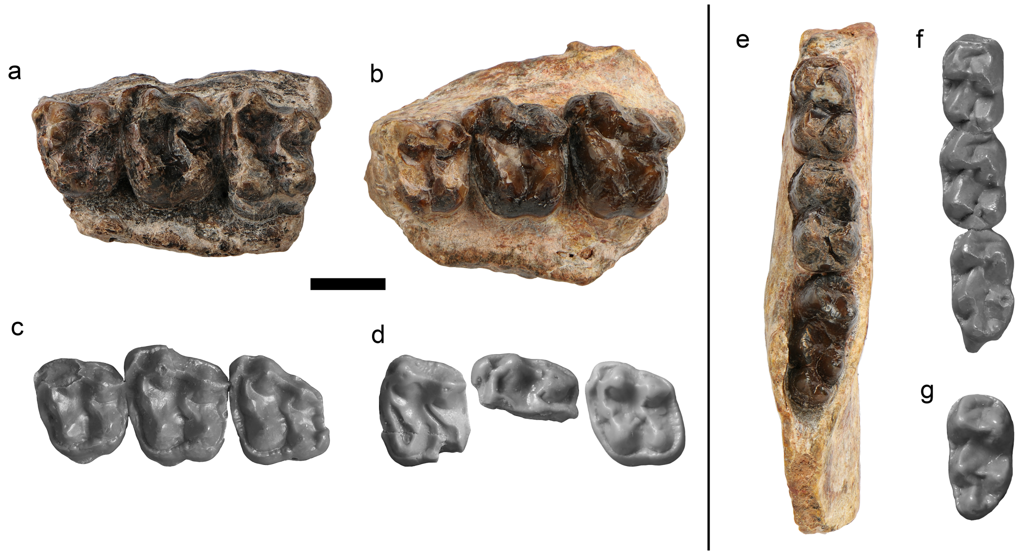

Supplementary Figure 1. Comparisons of the earliest Eocene equids and ceratomorph *Orientolophus*. a, *Erihippus tingae* gen. et sp. nov., left maxilla with DP4-M2 (IVPP V 5790); **b,** *Orientolophus hengdongensis*, right maxilla with DP4-M2 (reversed, IVPP V 5789, lectotype); **c,** *Sifrhippus sandrae*, left M1-3 (AMNH FM 127481, cast of UM 79889); **d,** *Cymbalophus cuniculus*, right M1 and left M2-3 (AMNH FM 119185 (reversed), 119186, and 119190, casts of NHMUK PV M29709, Ipswich Museum no. 1971.169, and NHMUK PV OR36569, lectotype); **e,** *E. tingae*, left mandible with m1-3, IVPP V 5789.1, holotype; **f,** *S. sandrae*, right m1-3 (reversed, AMNH FM 127483, cast of UM 83567, holotype); **g,** *C. cuniculus*, right m3 (reversed, AMNH FM 119191, cast of Ipswich Museum no. 1951.28.25). Scale: 5 mm.

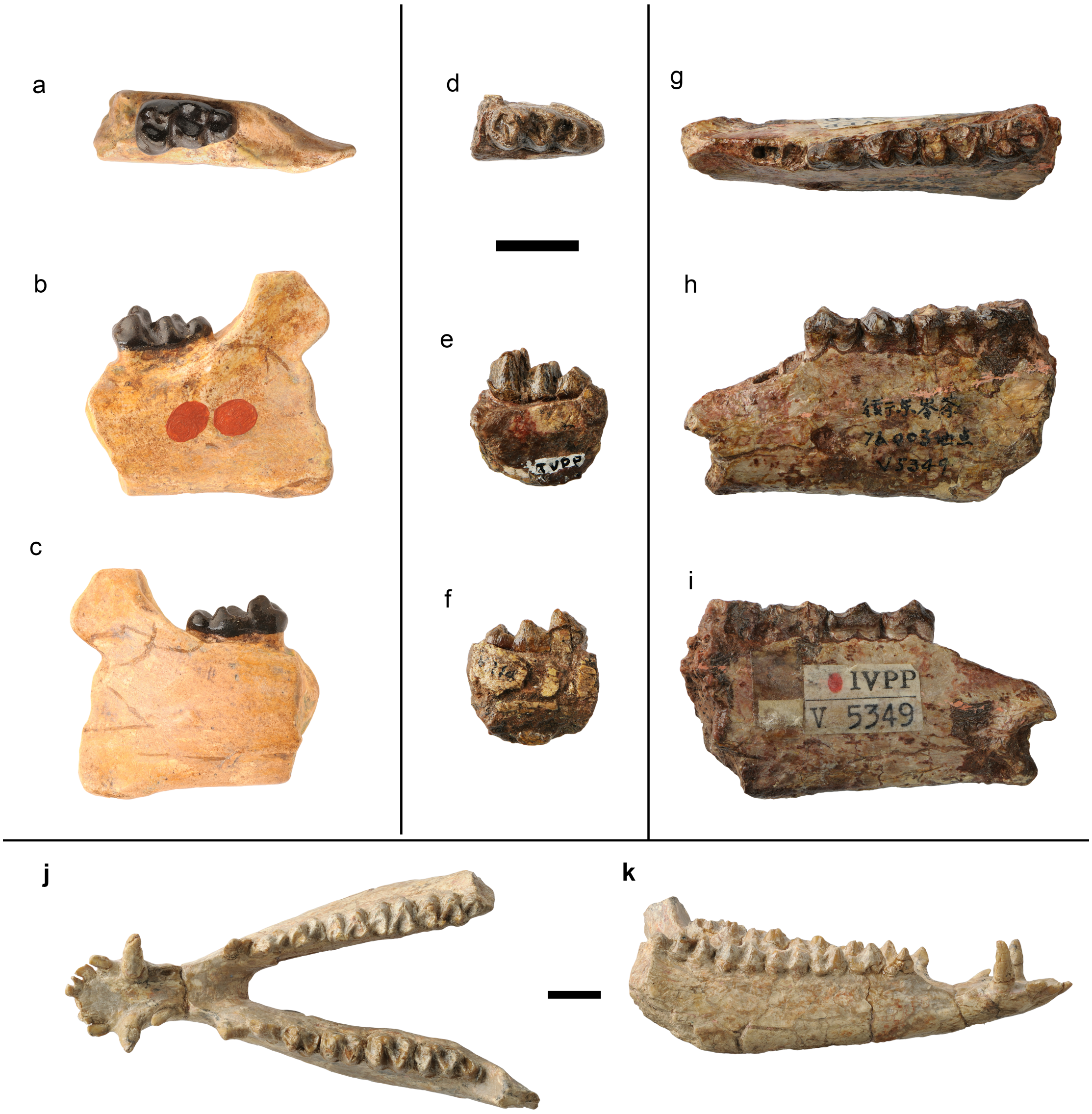

Supplementary Figure 2. Comparisons of the early Eocene ancylopods and brontotheres. a-f, *Protomoropus*? *hengyangensis* (Young, 1944), **a-c,** left mandible with m3 (IVPP V 214, cast, holotype) in occlusal (a), buccal (b), and lingual views (c); **d-f,** left mandible with m3 (IVPP V 7453) in occlusal (d), buccal (e), and lingual views (f); **g-i,** *Danjiangia lambdodon* sp. nov., left mandible with p3-m2 (IVPP V 5349, holotype) in occlusal (g), buccal (h), and lingual views (i); **j-k,** lower jaw of *Danjiangia pingi* (IVPP V 10842, holotype) in occlusal (j) and right buccal views (k). Scale: 1 cm.

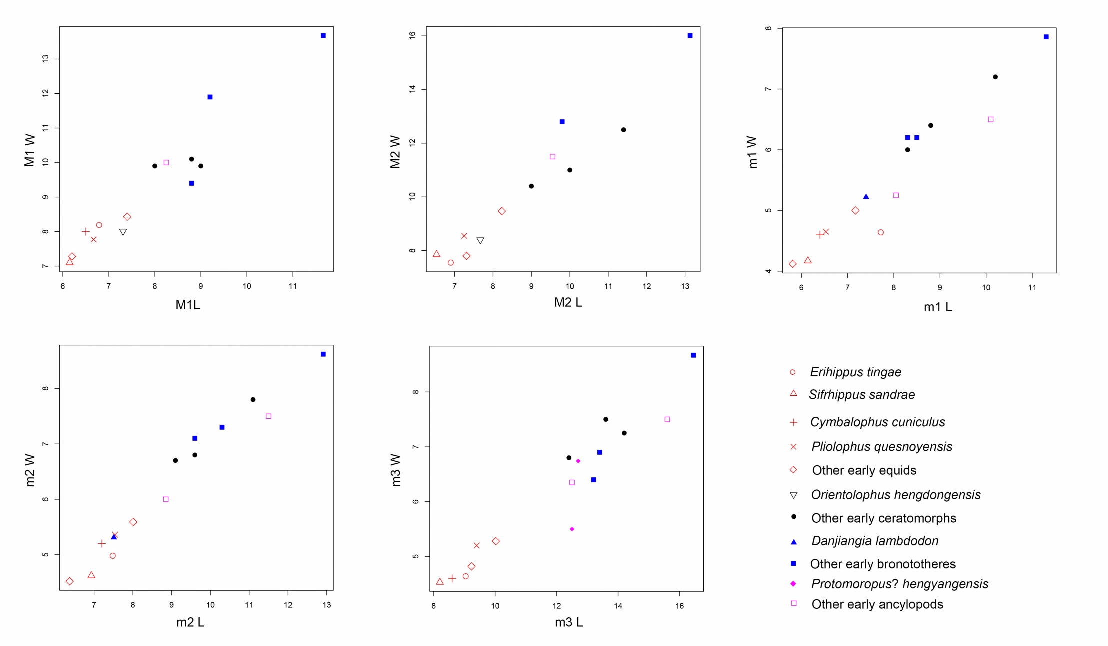

Supplementary Figure 3. Scatter plots for molar size of compared early perissodactyls. Red, black, blue, and magenta symbols represent early equids, ceratomorphs, brontotheres, and ancylopods, respectively. For raw data, refer to the Supplementary Table 1.

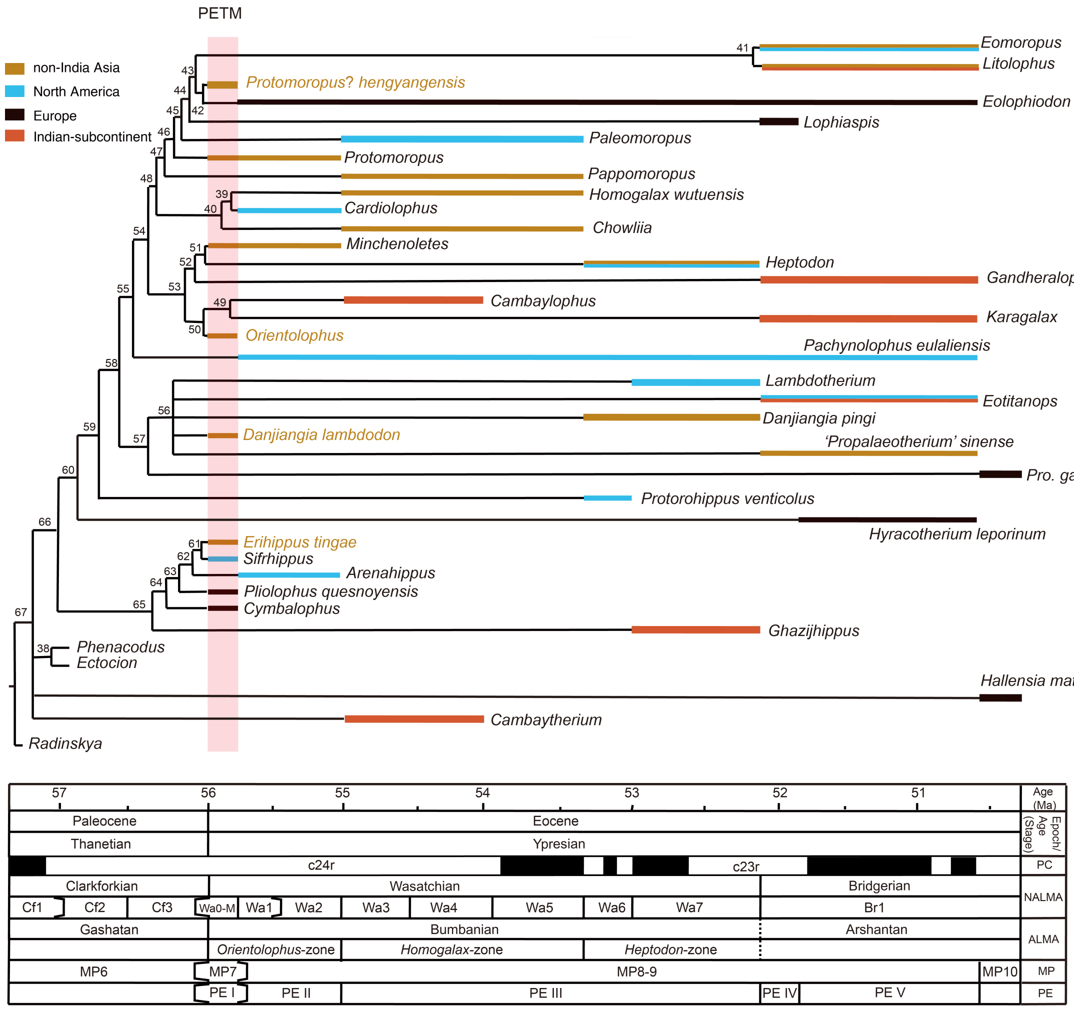

Supplementary Figure 4. The strict consensus tree of 63 equally most parsimonious trees and the distribution of selected taxa. The unambiguous synapomorphies at nodes are listed in the Supplementary Table 2. The bottom column showing Polarity Chron (PC), North American Land Mammal Ages (NALMA), Asian Land Mammal Ages (ALMA), European Reference Levels (MP), and Paleocene-Eocene transition (PE) modified from the sources[1-5].

Supplementary Table 1.

Measurements of *Erihippus*, *Orientolophus*, *Lambdodon* and other compared early perissodactyls. Measurements of *Sifrhippus* from Gingerich[6], *Pliolophus quesnoyensis* from the mean value according to Bronnert, et al.[7], *Arenahippus* from Kitts[8], *Chowliia* and *Paleomoropus* from Tong and Wang[9], *Cardiolophus* from Gingerich[10], *Danjiangia* from Wang[11], '*Propalaeotherium*' *sinense* from Zdansky[12], *Protomoropus* from Hooker and Dashzeveg[13]. The measurements of *Cymbalophus* were measured from the figures of Hooker[14]. The measurements of *Minippus* were measured from the casts of AMNH FM 4680 and NMMNH P9589. The measurements of *Lambdotherium* were from the casts of CM 62459 and CM 4963.

species	M1		M2		M3		m1		m2		m3	
	L	W	L	W	L	W	L	W	L	W	L	W
Erihippus	6.79	8.19	6.9	7.55	NA	NA	7.72	8.19	7.48	4.98	9.04	4.64
Sifrhippus	6.15	7.1	6.53	7.85	6.13	7.26	6.14	7.1	6.93	4.62	8.2	4.53
Cymbalophus	6.5	8	7.5	NA	6.5	7.5	6.4	8	7.2	5.2	8.6	4.6
Pliolophus quesnoyensis	6.67	7.77	7.25	8.55	7.8	8.6	6.53	7.77	7.54	5.36	9.4	5.2
Arenahippus	7.4	8.43	8.23	9.47	7.62	9.35	7.17	8.43	8.01	5.59	10.02	5.28
Minihippus	6.2	7.28	7.31	7.8	6.55	7.53	5.81	7.28	6.37	4.52	9.23	4.82
Orientolophus	7.31	8.01	7.67	8.4	NA	NA	NA	NA	NA	NA	NA	NA
Chowliia	8.8	10.1	11.4	12.5	10.1	12.5	10.2	10.1	11.1	7.8	14.2	7.25
Cardiolophus radinskyi	9	9.9	9	10.4	8.8	10	8.8	9.9	9.1	6.7	12.4	6.8
Cardiolophus radinskyi	8	9.9	10	11	8.8	11.1	8.3	9.9	9.6	6.8	13.6	7.5
Danjiangia lambdodon	NA	NA	NA	NA	NA	NA	7.4	NA	*7.51*	*5.31*	NA	NA
Proto.?	NA	NA	NA	NA	NA	NA	NA	NA	NA	NA	12.7	6.74
hengyangensis	NA	NA	NA	NA	NA	NA	NA	NA	NA	NA	12.5	5.5
Danjiangia	9.2	11.9	9.8	12.8	9.3	12.6	8.3	11.9	9.6	7.1	13.4	6.9
Pro. sinense	8.8	9.4	NA	NA	10.8	12.6	8.5	9.4	10.3	7.3	13.2	6.4
Lambdo-therium	11.66	13.68	13.13	16.01	13.31	15.92	11.3	13.68	12.91	8.62	16.45	8.67
Protomoropus gabuniai	8.25	10	9.55	11.5	10.2	11.7	8.05	10	8.85	6	12.5	6.35
Paleomoropus	NA	NA	NA	NA	NA	NA	10.1	NA	11.5	7.5	15.6	7.5

Supplementary Table 2.

Distribution of unambiguous synapomorphies (strict consensus tree, Supplementary Fig. 4)

Node	Characters and state
38	9(0), 10(0), 12(0), 14(2), 15(2), 17(0), 20(0), 22(0), 26(0), 29(0), 38(0), 39(0), 46(0), 57(0), 58(0), 66(0), 67(1)
66	4(1), 28 (1), 31(1), 35(1), 54(1), 58(1), 67(3)
60	14(1), 15(1)
59	8(1), 16(2), 18(0), 32(1), 41(1), 49(1), 66(0)
58	11(2), 28(2), 31(3), 42(2), 52(1), 58(3), 62(1)
55	1(0), 5(0), 19(1), 55(1)
54	14(0), 15(0), 18(2), 23(1), 30(0), 34(0), 38(0), 42(3)
48	8(0), 13(1), 44(1), 58(2)
40	7(2), 63(1)
39	56(1), 67(2)
47	18(1), 37(1)
46	16(3), 45(1)
45	13(3), 47(1), 62 (2)
44	65(2)
43	25(0), 27(1), 49(0)
41	18(0), 19(0), 44(0), 56(1), 61(2)
42	21(3)
53	4(2), 17(0), 36(1)
50	28(1), 63(1), 65(2)
49	25(0), 49(0)
52	3(2), 13(3)
51	1(2), 7(2), 21(2), 27(1)
57	4(0), 16(3), 17(2), 44(1)
56	1(3), 2(1), 14(2), 15(2), 23(1), 31(2), 34(0), 35(0), 49(2), 50(3), 58(2), 61(2), 62(2), 63(2), 65(2), 67(4)
65	3(1), 23(1), 52(1)
64	1(0), 5(0), 13(2), 67(1)
63	25(0)
62	42(4), 51(1), 55(1)
61	60(1), 61(1), 64(1)

Supplementary Note 1

Geological Setting

The Hengyang Basin is located in the south-central part of Hunan Province, China. It is roughly square with an area of about 5200 square kilometers[15]. Cretaceous rocks are widely distributed in the basin, whereas the early Paleogene deposits are mainly exposed in the northeastern part of the Basin. The early Eocene mammalian fossils were found from the Lingcha area, about 15 km southwest of Hengdong County. The research history of the "Red Beds" in Lingcha area has been summarized in detail by Ting[16].

The early Paleogene deposits in the Lingcha area form a syncline with an axis directed northeast[16]. The strata consist of the late Paleocene Limuping Formation with the lower Lingcha Fauna and the early Eocene Lingcha Formation with the upper Lingcha Fauna[17,18]. The Limuping Formation is predominantly purplish red silty mudstone with brownish purple, gray muddy siltstone, sandstone, and muddy dolomite[17]. The Lingcha Formation is predominantly brownish red silty mudstone with brownish purple/yellow silty sandstone and sandstone. The mudstone of the Lingcha Formation often bears more or less calcareous nodules[19]. Three sections have been measured from the Lingcha area[16,19]. Sections 1 and 2 are in the south limb of the syncline, whereas section 3 is in the north. Section 1 is measured from Pukuitang to Lingcha, and is about 555.4 meters thick[16]. The upper Lingcha Fauna was found between the 530 and 540 meters levels of the section[16]. Section 2 is west of Limuping and 16.8 meters thick, bearing the lower Lingcha Fauna in the middle[16]. Section 3 is measured from Tianzhifen to near Jixianwan, and is about 284.4 meters thick. The upper Lingcha Fauna was found between the 255 and 257 meters levels of the section[16]. Based on a combination of field observations, isotopic data, and paleomagnetic data, the 548 meters level of section 1 was correlated with 272 meters level of section 3, and the remainder of these sections can be also correlated on the basis of assuming equal sedimentation rates[16]. Thus, the fossil-bearing level of section

3 can be correlated from 531 to 533 meters of section 1, which is within the range of the upper Lingcha Fauna in section 1. Section 2 is correlated to the ~350 m level of section 1.

The composite section with all values projected on the section 1 clearly shows the Carbon Isotope Excursion at the Paleocene-Eocene boundary[16]. The Paleocene-Eocene boundary was placed at 516 meters, roughly equivalent to 240 m in section 3 that first produced the unusually low $\delta^{13}C$ value. Ting[16] pointed out that the Upper Lingcha Fauna occurs from about 15 meters above the Paleocene/Eocene boundary to the minimum carbon isotope value at 548 meters[3].

Supplementary Note 2

Comparisons of dental characters among *Erihippus*, *Orientolophus*, and other representatives of early equids[6,7,14,20] and tapiromorphs[9,10].

	Equidae					Tapiromorpha			
	Sifrhippus sandrae	*Cymbalophus cuniculus*	*Pliolophus quesnoyensis*	*Arenahippus grangeri*	*Erihippus tingae*	*Orientolophus*	*Chowliia*	*Cardiolophus*	*Homogalax*
**DP4**									
1. Paraconule and metaconule	Distinct	?	Distinct	?	Distinct	Weak or absent	?	?	Weak or absent
2. Metacone fold	Present	?	Present	?	Present	Absent	?	?	?
**M1-2**									
3. Parastyle	Small	Small	Small	Small	Small	Large	Large	Large	Large
4. Buccal side of paracone	Pinched mesiodistally	Generally convex	Generally convex	Generally convex	Pinched mesiodistally	Generally convex	Generally convex	Generally convex	Generally convex
5. Lingual side of paracone	Nearly flat	Convex	Convex or nearly flat	Convex	Nearly flat	Convex	Convex	Convex	Convex
6. Buccal rib of metacone	Weak	Weak	Weak	Weak	Strong	Strong	Weak	Weak	Weak
7. Paraconule and metaconule	Distinct	Distinct	Distinct	Distinct	Distinct	Weak or confluent	Weak or confluent	Weak or confluent	Weak or confluent
8. Position of paraconule	Midpoint of protoloph	Midpoint of protoloph	Midpoint of protoloph	Midpoint of protoloph	Midpoint of protoloph	Close to protocone	Close to protocone	Close to protocone	Close to protocone
9. Metacone fold	Present	Present	Present or absent	Absent	Present	Absent	Present	Present or absent	Present or absent

10. Lingual cingulum	Interrupted at hypocone	Complete	Complete or interrupted at hypocone	Complete	Absent	Restrict in the central valley	Restrict in the central valley	Restrict in the central valley	Restrict in the central valley
**m1-3**									
11. Protolophid	Notched	Unnotched	Notched	Notched	Notched	?	Notched	Notched	Notched
12. Paralophid orientation	Mesiolingual-ly	Mesially and slightly lingually	Mesially and slightly lingually	Mesially and slightly lingually	Mesially and slightly lingually	?	Mesially	Mesially	Mesially
13. Twinned metaconid	Present	Present	Present	Present	Absent	?	Present	Present	Present
14. Cristid obliqua orientation	Midpoint of protolophid	Buccal to midpoint	Midpoint of protolophid	Midpoint of protolophid	Buccal to midpoint	?	Buccal to midpoint	Buccal to midpoint	Buccal to midpoint
15. Postcristid/hypo-lophid of m1-2	Deeply notched	Deeply notched	Deeply notched	Deeply notched	Deeply notched	?	Nearly unnotched	Nearly unnotched	Nearly unnotched
16. Postcristid/Hypo-lophid of m3	Absent, entoconid isolated	Absent or rudimentary	Absent or weak	Absent, entoconid isolated	Absent, entoconid isolated	?	Shallowly notched	Unnotched	Unnotched
17. Posthypocristid of m3	Present, connecting hypoconulid	Present, connecting hypoconulid	Present, connecting hypoconulid	Present, connecting hypoconulid	Present, connecting hypoconulid	?	Absent	Absent	Absent
18. Hypoconulid lobe of m3	Narrow	Narrow	Narrow	Narrow or wide	Wide	?	Wide	Wide	Wide
19. Cuspid lingual to hypoconulid of m3	Present, lingually placed	Present, mesially placed	Absent or mesially placed	Present, lingually placed	Present, distally placed	?	Present, distally placed	Inconspi-cuous, distally placed	Inconspicu-ous, distally placed

Supplementary Note 3

Character list for the phylogenetic analysis

Our phylogenetic analysis was based on the matrix derived from the data of Hooker and Dashzeveg[13], which includes various early perissodactyls. We separated the upper and lower dental characters, which were sometimes treated as combined characters based on the occlusal relationships by Hooker and Dashzeveg [13]. *Lophocion* was excluded, because it only preserves upper molars[21]. The new matrix consists of 37 taxa and 70 characters with 15 new taxa and 11 new characters added.

Recently, the early Eocene ceratomorphs *Cambaylophus* and *Vastanolophus* have been reported from India[22,23]. *Cambaylophus* was considered to be a sister group of later *Gandheralophus* from Pakistan and both of them probably originated from *Orientolophus*-like taxa[22], whereas *Vastanolophus* was considered to be a basal helaletid[23]. Because *Vastanolophus* was only represented by a single m1 and a fragmentary p4, this taxon was excluded from the matrix. Early Eocene *Ghazijhippus*, known from the lower part of the upper Ghazij Formation in Pakistan, represent a basal perissodactyl similar to *Hallensia*[24]. Early Eocene *Gandheralophus* from the upper part of the upper Ghazij Formation is composed of two species: *G. minor* and *G. robustus*[25]; the former is closer to the ancestral morphotype of the genus and was selected for the matrix. *Pachynolophus eulaliensis* and *Pliolophus quesnoyensis* were chosen to represent their respective genera in the matrix of Hooker and Dashzeveg[13], because of their relatively earlier age and more basal phylogenetic position. Similarly, *Eolophiodon* was chosen to replace *Lophiodon*[26].

The following specimens and/or references were checked for the character coding.

Outgroup:

Radinskya yupingae[27,28], IVPP V 5255, new added taxon

Phenacodus [29], AMNH FM 15275

Ectocion[29], AMNH FM 16099, 16214

Cambaytherium[30]

Ingroup:

Hallensia matthesi[31,32], Halle XIV/3106 (cast AMNH FM 119184)

Hyracotherium leporinum[33], NHMUK PV M16336 (cast AMNH FM 27773)

Cymbalophus cuniculus[14,34], NHMUK PV OR36569 (cast AMNH FM 119190), NHMUK PV M29709 (cast AMNH FM 119185), NHMUK PV M14112 (cast AMNH FM 119187); Ipswich Museum 1971.169 (cast AMNH FM 119186), 1951.28.25 (cast AMNH FM 119191); IRSNB M 167 (cast AMNH FM 13759)

Sifrhippus sandrae[10], UM 83567 (cast AMNH FM 127483), 79889 (cast AMNH FM 127481), 83473 (cast AMNH FM 127482), 83615 (cast AMNH FM 127484), [6]

Cardiolophus radinskyi, UM 78915, 68548

Orientolophus[35], IVPP V 5789

Lambdotherium[36], AMNH FM 4863; CM 4963, 62459

Karagalax mamikhelensis[37]

Pachynolophus eulaliensis[38]

Pliolophus quesnoyensis[7]

Protomoropus gabuniai[13], PSS. 20-9 (cast AMNH FM 113856)

Homogalax wutuensis[9,39], IVPP V 2809

Lophiaspis[40]

Paleomoropus jepseni[41], YPM VPPU 13254

Litolophus gobiensis[42,43], IVPP V 16139, 16141, 16149

Eolophiodon[26]

Eomoropus[41,44], AMNH FM 5096; CM 3109

Heptodon [45,46], AMNH FM 4858

Ghazijhippus[24], new added taxon

Erihippus tingae[35], IVPP V 5790, 5789.1, new taxon

Arenahippus grangeri[20], AMNH FM 16134 (cast IVPP FV 446), new added taxon

Propachynolophus gaudryi[47], MSNL 6385 (cast AMNH FM 55904); MNHN

AL-5210 (cast AMNH FM 56600); NMB TS-628 (cast AMNH FM 80195), new added taxon

Chowliia laoshanensis[9], IVPP V 10739, 10740, new added taxon

Cambaylophus[22], new added taxon

Gandheralophus minor[25], GSP-UM 4710, 6770, new added taxon

Minchenoletes erlianensis[48], IVPP V 14683, 14686, 14694, new added taxon

Danjiangia lambdodon[18], IVPP V 5349, new taxon

Protomoropus? *hengyangensis*[16,49], IVPP V 214, 7453, new taxon

Pappomoropus taishanensis[9], IVPP V 10738, new added taxon

Danjiangia pingi[11], IVPP V 10842, new added taxon

'*Propalaeotherium*' *sinense*[12], PMUM 3446 (cast IVPP FV 720), 3013 (cast IVPP FV 710), 3444 (cast IVPP FV 719), No. 2 (cast IVPP FV 702), new added taxon

Eotitanops[50], CM 67793, 61941, 22442, new added taxon

Protorohippus venticolus[20], AMNH FM 4832, new added taxon

Characters

1. Degree of check teeth lophodonty: (0) general lophodonty with small, more or less confluent paraconule and metaconule; (1) weak lophodonty with relatively large paraconule and metaconule; (2) strong lophodonty without paraconule and metaconule; (3) lophodonty with a large paraconule but reduced or lack of metaconule. New character.
2. Upper molar preparaconule crista direction towards: (0) parastyle; (1) preparacrista, sometimes joining it; (2) preparacrista, constantly joining it. (Hooker and Dashzeveg, 2004, Ch. 2)
3. Upper molar paraconule situated related to the protocone: (0) distinctly more mesially; (1) scarcely more mesially; (2) paraconule reduced, situated at nearly the same buccolingual plane as the protocone. (Modified from Hooker and Dashzeveg, 2004, Ch. 3)

4. Occupation of upper molar facet 2A: (0) paraconule and part of preprotocrista; (1) only the paraconule; (2) facet 2A absent, with facet 2 and 3 nearly aligned. (Modified from Hooker and Dashzeveg, 2004, Ch. 3)
5. Molar preprotocrista: (0) unnotched; (1) notched (Modified from Hooker and Dashzeveg, 2004, Ch. 3)
6. 'Metastylid' separated from the metaconid: (0) metaconid rudimentarily twinned; (1) metaconid narrowly twinned; (2) metaconid widely twinned; (3) metaconid not twinned. (Modified from Hooker and Dashzeveg, 2004, Ch. 3)
7. Lower molar cristid obliqua: (0) absent; (1) straight; (2) bowed buccally (modified from Hooker and Dashzeveg, 2004, Ch. 4)
8. Lower molar protolophid: (0) notched; (1) shallowly indented, lophoid. (Hooker and Dashzeveg, 2004, Ch. 5)
9. Lower molar metastylid: (0) a prominent cuspule; (1) weak to lacking (Hooker and Dashzeveg, 2004, Ch. 6)
10. Position of upper molar metaconule: (0) on a line drawn between the metacone and hypocone; (1) (or its position if subsumed by metaloph) distinctly mesial of a line drawn between the metacone and hypocone (Modified from Hooker and Dashzeveg, 2004, Ch. 7)
11. m1-2 hypoconulid: (0) connecting the hypoconid; (1) connecting the middle of the hypoconid and entoconid (hypolophid); (2) small and appressed to the talonid; (3) large and isolated (Modified from Hooker and Dashzeveg, 2004, Ch. 7)
12. Upper molar metaconule: (0) not joined to hypocone by crest; (1) joined to the hypocone by crest (Hooker and Dashzeveg, 2004, Ch. 8)
13. Size of upper molar metaconule: (0) large; (1) size variable, some large, some small; (2) small (3) absent (Modified from Hooker and Dashzeveg, 2004, Ch. 9)
14. Upper molar centrocrista: (0) straight; (1) slightly flexed buccally; (2) sharply flexed buccally (Hooker and Dashzeveg, 2004, Ch. 10)
15. Upper molar mesostyle: (0) lacking; (1) small or variably developed; (2) large

(modified from Hooker and Dashzeveg, 2004, Ch. 11)

16. Lower molar buccal and lingual cusp outer walls converge at about: (0) 45°; (1) 20°; (2) 10°; (3) 5°. (Hooker and Dashzeveg, 2004, Ch. 12)
17. Lower molar metaconid buttress: (0) absent; (1) present, buccal in position; (2) present, lingual in position. (Hooker and Dashzeveg, 2004, Ch. 13)
18. Lower molar cristid obliqua attaches to trigonid: (0) nearer to metaconid than to protoconid; (1) midway between protoconid and metaconid; (2) nearer to protoconid than to metaconid (Hooker and Dashzeveg, 2004, Ch. 14)
19. Attachment of lower molar cristid obliqua on back wall of trigonid: (0) high; (1) low (Hooker and Dashzeveg, 2004, Ch. 15)
20. Lower molar entoconulid: (0) a prominent cuspule; (1) weak to lacking (Hooker and Dashzeveg, 2004, Ch. 16)
21. Lower molar paracristid, when slightly worn, makes angle to tooth long axis of: (0) 50°; (1) 40°; (2) 30°; (3) 20°; (4) 10° (Hooker and Dashzeveg, 2004, Ch. 17)
22. Lower molar paracristid with mesiobuccal angle: (0) rounded; (1) sharp or bulging (Hooker and Dashzeveg, 2004, Ch. 18)
23. Lower molar trigonid back wall: (0) shallow; (1) steep (Hooker and Dashzeveg, 2004, Ch. 19)
24. P1: (0) present; (1) absent (Hooker and Dashzeveg, 2004, Ch. 20)
25. M1-2: (0) as long as broad; (1) broader than long (Hooker and Dashzeveg, 2004, Ch. 21)
26. M3 hypocone: (0) absent; (1) present (Hooker and Dashzeveg, 2004, Ch. 22)
27. M1-2 postmetacrista: (0) short; (1) long (modified from Hooker and Dashzeveg, 2004, Ch. 23)
28. Upper molar parastyle: (0) small; (1) medium; (2) large (Hooker and Dashzeveg, 2004, Ch. 24)
29. Size of M3: (0) smaller than M2; (1) not smaller than M2 (modified from Hooker and Dashzeveg, 2004, Ch. 25)

30. Relative length of m3 hypoconulid: (0) as close to hypoconid as this is to entoconid; (1) more distant but closer to hypoconid than this is to protoconid; (2) as far or further from hypoconid than this is from protoconid. (Hooker and Dashzeveg, 2004, Ch. 26)
31. m3 hypoconulid forming: (0) simple terminal cusp; (1) distal margin of post-talonid lobe; (2) bearing a lobe with a circular basin; (3) bearing a lobe with a cuspule. (modified from Hooker and Dashzeveg, 2010, Ch. 27)
32. m3 hypolophid: (0) incomplete; (1) complete (Hooker and Dashzeveg, 2004, Ch. 28)
33. P3 metacone compared with paracone: (0) smaller; (1) same size (Hooker and Dashzeveg, 2004, Ch. 29)
34. P3: (0) trigon relatively narrow, protoloph weak, paraconule very small and poorly defined; (1) trigon broader, protoloph stronger, paraconule much larger and better defined, but smaller than protocone (modified from Hooker and Dashzeveg, 2004, Ch. 30)
35. p3 metaconid: (0) very small and poorly defined; (1) much larger and better defined, but smaller than protoconid (modified from Hooker and Dashzeveg, 2004, Ch. 30)
36. p3 paraconid: (0) weak and much lower than protoconid; (1) strong and approaching height of protoconid (Hooker and Dashzeveg, 2004, Ch. 31)
37. p1: (0) present; (1) absent (Hooker and Dashzeveg, 2004, Ch. 32)
38. Position of optic foramen related to anterior lacerate foramen: (0) significantly anteriorly; (1) close (Hooker and Dashzeveg, 2004, Ch. 33)
39. Navicular facet of astragalus: (0) convex; (1) saddle-shaped (Hooker and Dashzeveg, 2004, Ch. 34)
40. Astragalar canal: (0) present; (1) absent (Hooker and Dashzeveg, 2004, Ch. 35)
41. Upper molars: (0) with no metaloph; (1) with prehypocrista but without metacone fold; (2) metacone fold some joined to metaconule, some not; (3)

consistently joined to metaconule forming complete metaloph; (4) buccal end of metaloph (homologue of metacone fold) shifted mesially from metacone (modified from Hooker, 2010[51], Ch. 32)

42. Lower preultimate molar: (0) without hypolophid; (1) hypolophid complete, comprising equal buccal and lingual segments of former postcristid joining in middle at notch in front of hypoconulid; (2) buccal segment lengthened at expense of lingual segment with lingual hypoconulid; (3) equal buccal and lingual segments joined into strong unnotched loph, hypoconulid median; (4) hypolophid notched but complete like state 1, some with lingual segment broken; (5) hypolophid with lingual segment consistently broken (Hooker and Dashzeveg, 2004, Ch. 37)
43. Lower m1-2 distal cingulum lingual of hypoconulid: (0) absent; (1) present (Hooker and Dashzeveg, 2004, Ch. 38)
44. Lower molar hypolophid (or line drawn between hypoconid and entoconid), especially on m3: (0) transverse; (1) oblique (modified from Hooker and Dashzeveg, 2004, Ch. 39)
45. Distal lower molar metaconid compared to mesial metaconid: (0) larger than or equal to; (1) distal smaller than mesial (Hooker and Dashzeveg, 2004, Ch. 40)
46. Mesial crest of lower molar metaconid: (0) present; (1) absent (Hooker and Dashzeveg, 2004, Ch. 41)
47. Upper molar parastyle: (0) pointing essentially occlusally; (1) recurved strongly distally (Hooker and Dashzeveg, 2004, Ch. 42)
48. Position of M3 parastyle: (0) aligned with those of M1-2; (1) projecting strongly buccally in some individuals; (2) consistently projecting strongly buccally (Hooker and Dashzeveg, 2004, Ch. 43)
49. Upper molar metacone: (0) vertically implanted; (1) tilted lingually slightly; (2) tilted lingually markedly (Hooker and Dashzeveg, 2004, Ch. 44)
50. Upper molar paracone: (0) vertically implanted; (1) tilted buccally slightly; (2)

tilted buccally markedly; (3) tilted lingually (modified from Hooker and Dashzeveg, 2004, Ch. 45)

51. P4 postprotocrista: (0) present; (1) absent (Hooker and Dashzeveg, 2004, Ch. 46)
52. P4 metaconule: (0) present, large; (1) weak; (2) absent (Hooker and Dashzeveg, 2004, Ch. 47)
53. P4 premetaconule crista: (0) weak; (1) strong, high on ectoloph (Hooker and Dashzeveg, 2004, Ch. 48)
54. P4 metaconule and postprotocrista or its position: (0) distal of a line between metacone and protocone; (1) more mesial (Hooker and Dashzeveg, 2004, Ch. 49)
55. P3 postprotocrista: (0) absent; (1) faint; (2) strong (Hooker and Dashzeveg, 2004, Ch. 50)
56. P3 paracone and metacone: (0) well separated as on P4; (1) closer together; (2) very close (Hooker and Dashzeveg, 2004, Ch. 51)
57. Post P1 diastema: (0) as long as upper postcanine diastema; (1) shorter; (2) absent (Hooker and Dashzeveg, 2004, Ch. 52)
58. Post p1 diastema: (0) as long as lower postcanine diastema; (1) shorter; (2) longer; (3) absent (modified from Hooker and Dashzeveg, 2004, Ch. 53)
59. p2-3 diastema: (0) present; (1) absent (Hooker and Dashzeveg, 2004, Ch. 54)

New characters

60. Lingual side of upper molar paracone: (0) convex; (1) flat or slightly convex; (2) with a crista.
61. Buccal side of upper molar paracone: (0) rounded (or concial); (2) mesiodistally pinched; (2) generally flat with a crista.
62. Buccal surface of upper molar metacone: (0) distinctly convex; (1) slightly convex; (2) nearly flat.
63. Upper molar paraconule position: (0) midway between paracone and protocone; (1) closer to protocone; (2) closer to paracone.
64. Position of hypocone related to protocone on molars: (0) equally lingually placed

or more lingually; (1) slightly more buccally.

65. Upper molar lingual cingulum: (0) absent; (1) generally complete; (2) restricted in the central valley
66. A cuspule lingual to hypoconulid on m3: (0) absent; (1) present, distolingually placed and smaller; (2) present, mesiolingually placed; (3) present, distolingually placed and larger
67. Buccal branch of m3 hypoconulid lobe direction towards: (0) buccal branch absent; (1) hypoconid; (2) the point closer to hypoconid than to entoconid; (3) the midpoint of hypolophid; (4) entoconid.
68. Mastoid exposure orientation: (0) occipital; (1) lateral; (2) absent. (Modified from Holbrook, 2014[27], ch. 7)
69. Nasal shape: (0) posteriorly narrow; (1) posteriorly broad (Holbrook [52], 1999, C2)
70. Nasolacrimal contact: (0) present; (1) absent (Holbrook [52], 1999, C3)

MATRIX

```
         1         2         3         4         5         6         7
1234567890123456789012345678901234567890123456789012345678901234567890
-------------------------------------------------------------------------------
Radinskya
00000????0?1000????????011001???????????1?????00000000?????000000??110
Phenacodus
10001-1000000220000000000000000000000000000-0000000000000000000101000
Cambaytherium
1100100011300 00?1--1--0001001200000 00?100001-1--0011?00212100000030?1?
                                                   2
Hallensia matthesi
110011101101000?11113100110001001100011120100?000010001112000000220?10
Hyracotherium leporinum
12011110110101111101110011111121011???11?25000?00000001201?100001123?11
Cymbalophus
02110111111120031211311?1111101011100???2100010001?10100?1100100121???
```

Sifrhippus sanderi

0211011011012003111121 1?011111011000111240000000000110110?1?11101121???

1

Cardiolophus

021?012111211002121141101102103110100?114311010110011111021000102022 1?

1

Orientolophus

021?0????1?1200?????????1?01?????????????1???????10?????????000102?????

Lambdotherium

3100111111212222320010100110212211000 1?1112010100230101201-10222020411?

Ectocion

11001-10000002210100001010010000000000000010000-00000000000000000120101???

Karagalax

021203111121200202114 11?011111311001 0?112310-0000001 0110??110110203???

3

Pachynolophus eulaliensis

021?01111121211?1011110011001231110001??120001001001010011100101 1?3?1?

1 1 3

Pliolophus quesnoyensis

021?01101101200?1111211?0101121010100?111100010000010100?1100000121???

1

Protomoropus

32111210112110031111111?111210211???????3311110200021 1?????0210110 3???

Homogalax wutuensis

0211022011?12003121141 1?1?02?03110??????3311110?10011111???0011020 2???

Lophiaspis

32111211112130032111211?110210?11???????4311111212021112??1002112?????

Paleomoropus

32111????1?1300?????????11021????????????43????1211?????????002001?????

Litolophus

3211121111213002200111100112103111101?114310111200020121 1-11220110 3?1?

Eolophiodon

321?031111213 00?21113111011210311010 1???4301-10002021102--12021120?110

Eomoropus

3211121111213223200111 1?011210311010101143101112000211212-112201203210

Heptodon

22220321112130030211211111111031101100113310-0001101112023100 2-1103110

Ghazijhippus

1211111011?1000?11111110110210101 1??0???4??10?000001012011100001213?1?

Erihippus tingae

02110010110120021211311?1?011010????????3400-10?00?????????11001011???

1

Arenahippus

121101???101200??????1100101101011100?11240001000011011011100000121???

Propachynolophus gaudryi

1210121111210113200131O?110212311110O???12010100100101201310010010З???

Chowliia

021102201121100212114110110210311000O???431101001001111O?210011010З???

2

Cambaylophus

021?0????1?1200?????????01011????????????3?????0000?????????001102?????

Gandheralophus

0222031111213001021141I?1102103110I?0???3300-1001002111I?310000010?2???

Minchenletes

222203211121300302112111?1?121???????????4301-10010110I?????202-0??????

Danjiangia lambdodon

??????1?1?2???????00?2??????????????00????????1???????????????1???????????

Protomoropus? hengyangensis

?????2111??????1211130I??????221????????????111???????????????????03???

2 3 1

Pappamoropus

?????2101?2?????21111101??????231??101????31100?????????????21??????03???

Danjiangia pingi

3100121111212221200101101102122110000???12010110230101011210222120 4?1?

Propalaeotherium sinense

31001211112122232001111?11021221????????1211010?23?????????0222020З???

Eotitanops

300?11111121222320011110100202310000 0?111201-100230101201210222000 4???

Protorohippus venticolus

120110111101011210012100110111111110011115 00?100101001201110000010111?

Supplementary References

1 Vandenberghe, N., Hilgen, F. J. & Speijer, R. P. in *A Geologic Time Scale 2012* (eds F. M. Gradstein, J. G. Ogg, M. D. Schmitz, & G. M. Ogg) 855–921, (Elsevier, 2012).

2 Ting, S. Y. in *Dawn of the age of Mammals in Asia* (eds C. Beard & M R Dawson) 124–147 (Bull Carnegie Mus Nat Hist **34**, 1998).

3 Ting, S. *et al.* Asian early Paleogene chronology and mammalian faunal turnover events. *Vert PalAsiat* **49**, 1–28 (2011).

4 Hooker, J. J. in *Correlation of the Early Paleogene in Northwest Europe* (eds R. W. O'B. Knox, R. M. Corfield, & R. E. Dunay) 205–218 (Geol Soc Spec

Publ **101**, 1996).

5 Hooker, J. J. A two-phase Mammalian Dispersal Event across the Paleocene-Eocene transition. *Newsl Stratigr* **48**, 201–220 (2015).

6 Gingerich, P. D. New earliest Wasatchian mammalian fauna from the Eocene of northwestern Wyoming: composition and diversity in a rarely sampled high-floodplain assemblage. *Univ Mich Pap on Paleontol* **28**, 1–97 (1989).

7 Bronnert, C., Gheerbrant, E., Godinot, M. & Métais, G. A primitive perissodactyl (Mammalia) from the early Eocene of Le Quesnoy (MP7, France). *Hist Biol* **30**, 237–250, doi:10.1080/08912963.2017.1341502 (2017).

8 Kitts, D. B. American *Hyracotherium* (Perissodactyla, Equidae). *Bull Am Mus Nat Hist* **110**, 1–60 (1956).

9 Tong, Y. S. & Wang, J. W. Fossil mammals from the Early Eocene Wutu Formation of Shandong Province. *Palaeont Sin, New Ser C* **28**, 1–195 (2006).

10 Gingerich, P. D. Systematics and Evolution of Early Eocene Perissodactyla (Mammalia) in the Clarks Fork Basin, Wyoming. *Contrib Mus Paleont, Univ Mich* **28**, 181–213 (1991).

11 Wang, Y. A new primitive chalicothere (Perissodactyla, Mammalia) from the early Eocene of Hubei, China. *Vert PalAsiat* **33**, 138–159 (1995).

12 Zdansky, O. Die alttertiären Säugetiere Chinas nebst stratigraphischen Bemerkungen. *Palaeontol Sin*, *Ser C* **6**, 5–87 (1930).

13 Hooker, J. J. & Dashzeveg, D. The origin of chalicotheres (Perissodactyla, Mammalia). *Palaeontology* **47**, 1363–1386 (2004).

14 Hooker, J. J. A primitive Ceratomorph (Perissodactyla, Mammalia) from the Early Tertiary of Europe. *Zool J Linn Soc* **82**, 229–244, doi:Doi 10.1111/J.1096-3642.1984.Tb00545.X (1984).

15 Liu, X. & Fu, D. Y. Sedimentary facies and the tectonic development of the Hengyang Basin, Hunan Province. *Acta Geosci Sin* **8**, 13–36 (1986).

16 Ting, S. *et al.* in *Causes and consequences of globally warm climates in the*

early Paleogene (eds Scott L. Wing, Philip D. Gingerich, Birger Schmitz, & Ellen Thomas) 521–535 (Geol Soc Am Spec Pap **369**, 2003).

17 Tong, Y. S., Wang, Y. Q. & Li, Q. Subdivision of the Paleogene in Lingcha Area of Hunan Provice and Early Eocene Mammalian Faunas of China. *Geol Rev* **52**, 153–162 (2006).

18 Li, C. K., Qiu, Z. X., Yan, D. F. & Xie, S. H. Notes on some early Eocene mammalian fossils of Hengtung, Hunan. *Vert PalAsiat* **17**, 71–80 (1979).

19 Bowen, G. J. *et al.* Mammalian dispersal at the Paleocene/Eocene boundary. *Science* **295**, 2062–2065 (2002).

20 Froehlich, D. J. Quo vadis *Eohippus*? The systematics and taxonomy of the early Eocene equids (Perissodactyla). *Zool J Linn Soc* **134**, 141–256, doi:Doi 10.1046/J.1096-3642.2002.00005.X (2002).

21 Wang, J. W. & Tong, Y. S. A new phenacodontid condylarth (Mammalia) from the early Eocene of the Wutu Basin, Shandong. *Vert PalAsiat* **35**, 283–289 (1997).

22 Kapur, V. V. & Bajpai, S. Oldest South Asian tapiromorph (Perissodactyla, Mammalia) from the Cambay Shale Formation, western India, with comments on its phylogenetic position and biogeographic implications. *The Palaeobotanist* **64**, 95–103 (2015).

23 Smith, T. *et al.* First early Eocene tapiroid from India and its implication for the paleobiogeographic origin of perissodactyls. *Palaeovertebrata* **39**, 1–9, doi:10.18563/pv.39.2.e5 (2015).

24 Missiaen, P. & Gingerich, P. D. New Basal Perissodactyla (Mammalia) From The Lower Eocene Ghazij Formation of Pakistan. *Contrib Mus Paleont, Univ Mich* **32**, 139–160 (2014).

25 Missiaen, P. & Gingerich, P. D. New early Eocene tapiromorph perissodactyls from the Ghazij Formation of Pakistan, with implications for mammalian biochronology in Asia. *Acta Palaeontol Pol* **57**, 21–34, doi:Doi

10.4202/App.2010.0093 (2012).

26 Robinet, C., Remy, J. A., Laurent, Y., Danilo, L. & Lihoreau, F. A new genus of Lophiodontidae (Perissodactyla, Mammalia) from the early Eocene of La Borie (Southern France) and the origin of the genus *Lophiodon* Cuvier, 1822. *Geobios* **48**, 25–38, doi:10.1016/j.geobios.2014.11.003 (2015).

27 Holbrook, L. T. On the skull of *Radinskya* (Mammalia) and its phylogenetic position. *J Vert Paleont* **34**, 1203–1215, doi:10.1080/02724634.2014.854249 (2014).

28 McKenna, M. C., Chow, M., Ting, S. & Luo, Z. in *The evolution of perissodactyls* (eds D. R. Prothero & R. M. Schoch) 24–36 (Oxford University Press, 1989).

29 Thewissen, J. G. M. Evolution of Paleocene and Eocene Phenacodontidae (Mammalia, Condylarthra). *Univ Mich Pap on Paleontol* **29**, 1–107 (1990).

30 Rose, K. D. *et al.* Early Eocene fossils suggest that the mammalian order Perissodactyla originated in India. *Nat Commun* **5**, 1–9, doi:10.1038/ncomms6570 (2014).

31 Franzen, J. L. & Haubold, H. Ein neuer condylarthre und ein tillodontier (Mammalia) aus dem Mitteleozän des Geiseltales. *Palaeovertebrata* **16**, 35–53 (1986).

32 Franzen, J. E. *Hallensia* (Mammalia, Perissodactyla) aus Messel und dem Parisen Becker sowie Nachtrige aus dem Geiseltal. *Bull Inst Sci Nat Belg (Sci Terre)* **60**, 175–201 (1990).

33 Hooker, J. J. The beginning of the equoid radiation. *Zool J Linn Soc* **112**, 29–63 (1994).

34 Missiaen, P., Quesnel, F., Dupuis, C., Storme, J.-Y. & Smith, T. The earliest Eocene mammal fauna of the Erquelinnes Sand Member near the French-Belgian border. *Geol Belg* **16**, 262–273 (2013).

35 Ting, S. Y. A preliminary report on an Early Eocene mammalian fauna from

Hengdong, Hunan Province, China. *Kaupia* **3**, 201–207 (1993).

36 Bonillas, Y. The Dentition of *Lambdotherium*. *J Mammal* **17**, 139–142 (1936).

37 Maas, M. C., Hussain, S. T., Leinders, J. J. M. & Thewissen, J. G. M. A new isectolophid tapiromorph (Perissodactyla, Mammalia) from the Early Eocene of Pakistan. *J Paleont* **75**, 407–417, doi:10.1666/0022-3360(2001)075<0407:anitpm>2.0.co;2 (2001).

38 Danilo, L. *et al.* A New Eocene Locality in Southern France Sheds Light on the Basal Radiation of Palaeotheriidae (Mammalia, Perissodactyla, Equoidea). *J Vert Paleont* **33**, 195–215 (2013).

39 Chow, M. C. & Li, C. K. *Homogalax* and *Heptodon* of Shantung. *Vert PalAsiat* **9**, 15-21 (1965).

40 Savage, D., Russell, D. & Louis, P. Ceratomorpha and Ancylopoda (Perissodactyla) from the Lower Eocene Paris Basin, France. *Univ Calif Pub Geol Sci* **66**, 1–38 (1966).

41 Radinsky, L. B. *Paleomoropus,* a new early Eocene chalicothere (Mammalia, Perissodactyla), and a revision of Eocene chalicotheres. *Am Mus Novit* **2179**, 1–28 (1964).

42 Bai, B., Wang, Y. Q. & Meng, J. New craniodental materials of *Litolophus gobiensis* (Perissodactyla, "Eomoropidae") from Inner Mongolia, China, and phylogenetic analyses of Eocene chalicotheres. *Am Mus Novit* **3688**, 1–27 (2010).

43 Colbert, E. H. Chalicotheres from Mongolia and China in the American Museum. *Bull Am Mus Nat Hist* **67**, 353–387 (1934).

44 Osborn, H. F. *Eomoropus*, an American Eocene chalicothere. *Bull Am Mus Nat Hist* **32**, 261–274 (1913).

45 Radinsky, L. B. Evolution of the Tapiroid skeleton from *Heptodon* to *Tapirus*. *Bull Mus Comp Zool* **134**, 69–106 (1965).

46 Radinsky, L. Origin and early evolution of North American Tapiroidea. *Bull*

Peabody Mus Nat Hist **17**, 1–106 (1963).

47 Savage, D. E., Russell, D. E. & Louis, P. European Eocene Equidae (Perissodactyla). *Univ Calif Pub Geol Sci* **56**, 1–94 (1965).

48 Wang, Y. *et al.* Early Eocene perissodactyls (Mammalia) from the upper Nomogen Formation of The Erlian Basin, Nei Mongol, China. *Vert PalAsiat* **49**, 123–140 (2011).

49 Young, C.-C. Note on the first Eocene mammal from South China. *Am Mus Novit* **1268**, 1–3 (1944).

50 Osborn, H. F. Titanotheres of ancient Wyoming, Dakota, and Nebraska. *Monogr U S Geol Surv* **55**, 1–894 (1929).

51 Hooker, J. J. The mammal fauna of the early Eocene Blackheath Formation of Abbey Wood, London. *Monogr Palaeontogr Soc* **164**, 1–157 (2010).

52 Holbrook, L. T. The phylogeny and classification of Tapiromorph Perissodactyls (Mammalia). *Cladistics* **15**, 331–350 (1999).
